# *APOE* and plasma AD biomarkers: The role of genetic ancestry in Hispanics/Latinos

**DOI:** 10.1002/alz.71213

**Published:** 2026-03-12

**Authors:** Caitlin Cheung, Natasha Z. Anita, Paola Filigrana, Myriam Fornage, Kevin A. Gonzalez, Linda C. Gallo, Carmen R. Isasi, Robert C. Kaplan, Xihao Li, Freddie Márquez, Humberto Parada, Krista M. Perreira, Alberto R. Ramos, Tatjana Rundek, Wassim Tarraf, Fernando D. Testai, Charles DeCarli, Hector M. González, Tamar Sofer

**Affiliations:** ^1^ Department of Biostatistics Harvard T.H. Chan School of Public Health Boston Massachusetts USA; ^2^ CardioVascular Institute (CVI) Beth Israel Deaconess Medical Center Boston Massachusetts USA; ^3^ Department of Neurosciences University of California San Diego La Jolla California USA; ^4^ Department of Epidemiology & Population Health Albert Einstein College of Medicine Bronx New York USA; ^5^ Brown Foundation Institute of Molecular Medicine McGovern Medical School University of Texas Health Science Center at Houston Houston Texas USA; ^6^ Human Genetics Center Department of Epidemiology School of Public Health The University of Texas Health Science Center at Houston Houston Texas USA; ^7^ Department of Psychology San Diego State University San Diego California USA; ^8^ Department of Pediatrics Albert Einstein College of Medicine Bronx New York USA; ^9^ Division of Public Health Sciences Fred Hutchinson Cancer Center Seattle Washington USA; ^10^ Department of Biostatistics University of North Carolina at Chapel Hill Chapel Hill North Carolina USA; ^11^ Department of Genetics University of North Carolina at Chapel Hill Chapel Hill North Carolina USA; ^12^ Division of Epidemiology and Biostatistics School of Public Health San Diego State University San Diego California USA; ^13^ Department of Social Medicine University of North Carolina School of Medicine Chapel Hill North Carolina USA; ^14^ Department of Neurology University of Miami, Miller School of Medicine Miami Florida USA; ^15^ Department of Healthcare Sciences Institute of Gerontology Wayne State University Detroit Michigan USA; ^16^ Department of Neurology and Rehabilitation University of Illinois Chicago College of Medicine Chicago Illinois USA; ^17^ Department of Neurology University of California Davis Sacramento California USA; ^18^ Department of Medicine Harvard Medical School Boston Massachusetts USA

**Keywords:** Alzheimer's disease, amyloid/tau/neurodegeneration framework, apolipoprotein E, genetic ancestry, Hispanics/Latinos, plasma biomarkers

## Abstract

**BACKGROUND:**

Apolipoprotein E (*APOE*) alleles are well‐established genetic risk factors for Alzheimer's disease (AD), but their effects on AD biomarkers (amyloid beta [Aβ]42/40, phosphorylated tau [p‐tau]181, neurofilament light chain [NfL], and glial fibrillary acidic protein [GFAP]) may vary across populations due to ancestry‐, age‐, and sex‐related differences. We hypothesized that these effects vary across Hispanic/Latino background groups with distinct ancestral admixture.

**METHODS:**

We analyzed ε2 and ε4 allele associations with AD biomarkers using survey‐weighted linear regression models, adjusting for demographic covariates. Secondary analyses examined genetic analysis group‐ and ancestry‐specific effects.

**RESULTS:**

ε4 was associated with lower Aβ42/40 and higher p‐tau181and GFAP levels, but not with NfL, suggesting its role in Aβ and tau deposition and neuroinflammation. ε4 associations were stronger in those with higher European and lower African ancestry.

**DISCUSSION:**

These findings expand on prior studies suggesting that genetic ancestry modifies *APOE*‐associated AD risk in Hispanic/Latino populations and highlight the importance of capturing ancestry‐based heterogeneity in AD biomarker research.

## Highlights

- The apolipoprotein E ε4 allele is associated with amyloid beta (Aβ)42/40, phosphorylated tau (p‐tau)181, and glial fibrillary acidic protein (GFAP) in US Hispanics/Latinos.
- ε4 effects on Aβ42/40, p‐tau181, and GFAP are strongest in Cubans, Mexicans, and Puerto Ricans.
- Global European and African ancestries modify the ε4 association with p‐tau181.
- Findings highlight the importance of including diverse groups in biomarker studies.

## 1 BACKGROUND

Over the past 50 years, advances in Alzheimer's disease and related dementias (ADRD) research have transformed detection methods, enabling earlier and more precise diagnosis. Historically, diagnosis relied on *post mortem* identification of amyloid beta (Aβ) plaques and tau‐containing neurofibrillary tangles.^1^, ^2^ Modern approaches instead use the ATN framework, which measures amyloid (A), tau (T), and neurodegeneration (N) biomarkers through cerebrospinal fluid (CSF) and positron emission tomography (PET) imaging. Recent extensions to this framework include neuroinflammatory (I) biomarkers, forming the ATN(I) model.^3^ Specific cut‐points detect pathology where amyloid levels are decreased and tau, neurodegeneration, and neuroinflammatory levels are increased in diseased individuals.^4^, ^5^ These markers often indicate disease 15 to 20 years before clinical symptoms emerge,^6^ making the ATN(I) framework valuable for early detection. However, CSF extraction and imaging remain invasive and expensive, prompting exploration of plasma biomarkers as a more accessible solution.^6^, ^7^ Plasma ATN(I) biomarkers show moderate‐to‐strong correlations with their CSF counterparts,^8^, ^9^ are associated with amyloid‐ and tau PET positivity,^8^, ^10^, ^11^ and show promise for diagnostic performance across preclinical and clinical Alzheimer's disease (AD) stages, particularly when used in combination.^8^, ^9^, ^12^, ^13^ In this study, we use plasma Aβ42/40 (A), phosphorylated tau (p‐tau)181 (T), NfL (N), and GFAP (I) as our ATN(I) biomarkers.

**RESEARCH IN CONTEXT**

**Systematic Review**: The authors reviewed the literature using traditional sources. Prior work links apolipoprotein E (*APOE*) alleles to plasma amyloid/tau/neurodegeneration (ATN) biomarker levels, but few studies have examined these relationships in Hispanic/Latino populations or considered genetic ancestry. Relevant publications are appropriately cited.

**Interpretation**: Our findings show that *APOE* ε4 is associated with select plasma ATN biomarkers—lower amyloid beta 42/40 and higher phosphorylated tau 181 and glial fibrillary acidic protein—in US Hispanics/Latinos. For ε2, associations were not significant overall but showed variation across specific Hispanic/Latino background groups, suggesting ancestry‐related heterogeneity. These results confirm the distinctive impact of *APOE* alleles in a genetically diverse and understudied population.

**Future Directions**: Future studies should examine how genetic ancestry and other environmental, lifestyle, and socioeconomic factors influence *APOE*–biomarker associations and Alzheimer's disease risk. Longitudinal and omics studies could help clarify underlying mechanisms and potential clinical implications.

Increased age, female sex, and presence of the apolipoprotein E (*APOE*) ε4 allele are well‐established risk factors of ADRD, with additional contributions from other genetic variants, cardiometabolic conditions, and environmental and lifestyle factors such as education, diet, and exercise.^3^, ^14^, ^15^ The *APOE* gene encodes apoE, a glycoprotein that plays a key role in cholesterol regulation and lipid metabolism. This gene has three common alleles—ε2, ε3, and ε4—that have distinct effects on late‐onset AD risk: ε4 increases risk while ε2 decreases risk compared to the more common ε3 allele (≈ 80% globally).^16^ However, these associations can vary significantly across populations,^17^, ^18^, ^19^ particularly among understudied groups.

Hispanics/Latinos are an understudied group, with <5% representation in neuroimaging studies despite facing disproportionately higher rates of ADRD.^20^ Emerging evidence suggests that the relationship between *APOE* and AD is weaker among Hispanic/Latino individuals than other populations. For instance, Belloy et al.^21^ reported consistently weaker *APOE–*AD effect sizes among Hispanic/Latino individuals (ε4 vs. ε3: odds ratio [OR] = 1.90, 95% confidence interval [CI] = [1.70, 2.13]; ε2 vs. ε3: OR = 0.90, 95% CI = [0.74, 1.10]) than among non‐Hispanic White individuals (ε4 vs. ε3: OR = 3.48, 95% CI = [3.32, 3.64]; ε2 vs. ε3: OR = 0.55, 95% CI = [0.50, 0.60]). These discrepancies may be influenced by the high genetic diversity of the Hispanic/Latino population, who exhibit admixture from African, European, and Amerindian ancestries. Indeed, differences in both *APOE*–ADRD associations^22^, ^23^, ^24^ and ATN–ADRD associations^1^, ^25^, ^26^, ^27^ have been observed not only between racial/ethnic groups but also among Hispanic/Latino background groups with distinct ancestral compositions. Prior analyses have suggested that Amerindian ancestry may attenuate ε4‐related cognitive decline,^28^ although recent studies report the opposite pattern, with larger ε4–ADRD effect sizes in groups with higher Amerindian ancestry.^23^ Distinct genetic ancestries among Hispanic/Latino groups may also lead to distinct biomarker patterns,^29^, ^30^ yet direct comparisons of ATN(I) biomarkers and *APOE* effects across Hispanic/Latino background groups are lacking. This study addresses this gap by analyzing associations between *APOE* and plasma ATN(I) biomarkers across diverse Hispanic/Latino backgrounds to better understand ancestry‐related influences on ADRD risk.

Using data from the Hispanic Community Health Study/Study of Latinos (HCHS/SOL), we hypothesized that ε4 is associated with decreased plasma amyloid (Aβ42/40) and increased tau (p‐tau181), neurodegenerative (NfL), and neuroinflammatory (GFAP) biomarkers, consistent with its known role in ADRD pathogenesis, while ε2 shows the opposite pattern. We further hypothesized that genetic ancestry modifies these associations, leading to distinct biomarker profiles across Hispanic/Latino background groups. Given mixed prior evidence, examining these *APOE*–ATN(I) relationships across diverse Hispanic/Latino backgrounds may enhance biomarker diagnostics and refine ADRD risk predictions for groups at high biological risk.

## 2 METHODS

### 2.1 The Study of Latinos–Investigation of Neurocognitive Aging

The HCHS/SOL is a large‐scale longitudinal study of self‐identified Hispanic/Latino individuals from diverse backgrounds. The HCHS/SOL study design has been previously described.^31^ Between 2008 and 2011, the study recruited 16,415 individuals aged 18 to 74 across four US sites (Miami, San Diego, Chicago, and the Bronx). Visit 1 included comprehensive clinical assessments, such as anthropometry, blood pressure, biospecimen collection, and neurocognitive testing for middle‐aged and older adults (*n* = 9652), focusing on memory, verbal fluency, and processing speed.^32^ Visits 2 (2014–2017) and 3 (2020–2024) followed 11,623 and 9864 participants, respectively, with abbreviated protocols.^33^

The Study of Latinos–Investigation of Neurocognitive Aging (SOL‐INCA) ancillary study was initiated at HCHS/SOL Visit 2 to investigate midlife health, cognitive aging, and resilience among diverse Hispanic/Latino populations aged ≥50 years with baseline cognitive data.^32^ ATN(I) biomarkers were measured in the plasma of SOL‐INCA participants, as described in the next section. Of the 6377 SOL‐INCA participants, those with missing *APOE* genotype data, incomplete biomarker measurements, or extreme biomarker outliers were excluded, resulting in an analytic sample of 6118 individuals. All participants provided informed consent in their preferred language (English or Spanish) for using genetic and non‐genetic data. The study received approval from the institutional review boards at all collaborating institutions.

### 2.2 Measurement of ATN(I) biomarkers

We analyzed five key biomarkers associated with ADRD: Aβ40, Aβ42, p‐tau181, NfL, and GFAP. Plasma biomarker levels were measured in blood samples collected after an overnight fast as part of the SOL‐INCA study, as previously described.^34^ A single molecule array (Simoa) technique on the Quanterix Simoa HD‐X Analyzer was used to measure plasma Aβ40, Aβ42, NfL, and GFAP using the Neurology 4‐Plex E Advantage Kit. A subset of NfL samples was measured with the Simoa NF‐light Advantage Kit, and all p‐tau181 samples were measured with the Simoa pTau181 Advantage V2 Kit. Blood was centrifuged within 15 minutes of collection, and ethylenediaminetetraacetic acid plasma was aliquoted into 2.0 mL polypropylene screw‐cap vials. Samples were processed and stored at –70°C until analysis to preserve biomarker integrity. Reproducibility of measurements was evaluated by retesting a subsample using blinded duplicates; all coefficients of variation were <11.3%. Censored data, including those samples with values that were out of range or had insufficient protein to measure, were excluded from the study.

In both primary and secondary analyses, Aβ biomarker levels were assessed using the Aβ42 to Aβ40 concentration ratio (Aβ42/40), which has been shown to outperform Aβ42 concentration alone in identifying AD in both CSF^35^ and plasma.^36^ This ratio helps account for individual variability in Aβ production and clearance.^7^ Tau pathology, neurodegeneration, and neuroinflammation were assessed with p‐tau181, NfL, and GFAP biomarkers, respectively. Coefficients of variation (CV%) for plasma AD biomarkers are shown in Table S1 in supporting information. Within‐study CVs ranged from 20.5% (Aβ40) to 47.8% (NfL), while Quanterix‐reported inter‐assay CVs ranged from 3.7% (Aβ42) to 10.3% (NfL),^37^ indicating good assay precision but expected biological variability across biomarkers. Plasma biomarkers demonstrated weak to moderate pairwise correlations, with the strongest correlations observed between NfL and GFAP (*r* = 0.51) and p‐tau181 and NfL (*r* = 0.39; Figure S1 in supporting information).

### 2.3 Genetic characterization

Genetic characterization and quality control protocols have been previously described.^38^, ^39^ Genotyping for HCHS/SOL participants was performed using an Illumina custom array that included ancestry‐informative markers and single‐nucleotide polymorphisms (SNPs) from the 1000 Genomes phase 1 reference panel. Quality control steps included checks for genotyping accuracy, population structure, and relatedness.

The HCHS/SOL sample contains considerable familial relatedness, consistent with its household‐ and community‐based sampling design. As previously described,^39^ relatedness was evaluated using kinship coefficients (KCs) derived from KING‐robust, PC‐AiR, and PC‐Relate, which identified first‐ and second‐degree relatives (204 parent–offspring trios, 1042 parent–offspring duos, and 699 sibling pairs) and additional more distant relatives. Principal components (PCs) were computed using PC‐AiR to capture ancestry‐related population structure. Together, these measures quantify familial structure within the cohort.

Participants were classified into one of six “genetic analysis groups”—Central American, Cuban, Dominican, Puerto Rican, Mexican, and South American—based on both self‐reported background and genetic similarity. Genetic analysis groups were derived as hyper‐ellipsoids in the genetic PC space based on participants’ self‐identified Hispanic/Latino background. These genetically informed groups serve to reduce genetic heterogeneity within self‐identified Hispanic/Latino background groups and improve statistical power for genetic analysis by including all participants, including those who self‐reported as having more than one or other heritage. These groups are defined strictly for analytic purposes and do not imply any social, cultural, or biological categorization.^40^

*APOE* genotyping was conducted in the SOL‐INCA study to investigate ADRD, including cognitive aging, mild cognitive impairment (MCI), and dementia. SNPs rs429358 and rs7412 were genotyped using TaqMan assays, with polymerase chain reaction (PCR) amplification and genotype calling performed using standard real‐time PCR methods.^41^ Allele frequencies of these SNPs were consistent with the Hardy–Weinberg equilibrium within each genetic analysis group.^41^

### 2.4 Global ancestry inference

Global ancestry inference for the HCHS/SOL dataset has been previously described.^39^ To summarize, global ancestry proportions were derived from a supervised ADMIXTURE^42^ model (k = 3; African, Amerindian, and European). ADMIXTURE was trained using reference samples from the Human Genome Diversity Project (HGDP)^43^ and 1000 Genomes Projects^44^ after linkage disequilibrium pruning of 92,992 autosomal SNPs from 440,908 common genotyped variants. Reference samples with ≥90% ancestry from one inferred cluster, consisting of 101 African, 49 Amerindian, and 176 European individuals, were used for supervised analysis of 10,642 unrelated HCHS/SOL participants (relatedness threshold KC < 0.044). Participants were projected onto the learned structure to obtain global ancestry proportions, which were used in the current analyses.

### 2.5 Local ancestry inference

Local ancestry inference for the HCHS/SOL dataset has been previously described.^45^ Briefly, local ancestry was inferred using RFMix v1.5.4 with the PopPhased option and a minimum node size of 5, following recommended parameters. Genotype phasing was conducted using Beagle 4.0^46^ using intersection of SNPs from the Illumina Omni 2.5 M array and reference panels from HGDP and the 1000 Genomes Project.^43^, ^44^ After intersecting SNPs across datasets, ≈ 420,000 markers remained for local ancestry inference. ADMIXTURE analysis (k = 4, corresponding to African, European, Amerindian, and East Asian ancestral clusters) was used to estimate genome‐wide ancestry proportions for the purpose of defining reference panels for local ancestry inference. Individuals with ≥90% inferred ancestry from a single cluster were designated as reference samples representing European, West African, or Amerindian ancestral populations; East Asian reference individuals were excluded from this designation. This resulted in 195 West African, 63 Amerindian, and 527 European reference individuals.

For the present analysis, local ancestry at the *APOE* locus (chromosome 19q13.32, positions 44,905,796–44,909,393 based on hg38 coordinates) was extracted and coded as the number of African, Amerindian, or European ancestry copies (0, 1, or 2). Regression models analyzing local ancestry included global African and Amerindian ancestry proportions as covariates (with European as the reference) to account for genome‐wide admixture and reduce confounding.

### 2.6 Statistical analysis

We summarized demographic characteristics, biomarker levels, and *APOE* allele distributions using descriptive statistics for the full analytic dataset and stratified by genetic analysis group. To account for batch effects introduced by the 96‐well plates, we adjusted the biomarker levels by applying a linear mixed model (LMM) that included random effects for plate number and plate position. Adjusted biomarker values were used in subsequent analyses to minimize confounding due to batch effects. Extreme outliers in batch‐corrected biomarker values, defined as residuals falling more than ±3 times the interquartile range (IQR) beyond the first and third quantiles,^47^ were identified and removed to ensure the relative normality of residuals. We examined the distributions of plasma ATN(I) biomarker values (Aβ42/40, p‐tau181, NfL, GFAP) using visualization and descriptive statistics. No transformations were applied, as they did not noticeably improve normality. Residuals from survey‐weighted regression models were approximately normally distributed after batch adjustment and outlier removal (Figure S2 in supporting information).

To assess associations between ε2 and ε4 alleles and ATN(I) biomarker levels using survey‐weighted linear regression models (with ε3 as the reference allele), we used a complex survey design implemented in the R “survey” package, using a “gaussian” family for continuous variables. This approach accounted for stratification, clustering, and probability weighting in HCHS/SOL, enabling appropriate generalizations to the target US Hispanic/Latino population. The survey design also accounts for correlation between participants due to household‐ or community‐level relatedness, characterizing the HCHS/SOL target population while providing adjustment for non‐independence without explicitly modeling KCs in these analyses. Analyses were performed using an additive inheritance mode, in which allele counts per individual were coded as 0, 1, or 2 based on the number of copies of a given *APOE* allele. Individuals carrying both ε2 and ε4 alleles (≈ 1% of the total sample) were assigned a value of 1 for each allele without corrections for offsetting effects. To allow for direct comparison of effect sizes across biomarkers with differing units and scales, all regression coefficients were standardized by dividing by the survey‐weighted standard deviation (SD) of each biomarker, calculated from the overall analytic cohort. These same SDs were applied in all secondary analyses to maintain consistency.

Three nested regression models were evaluated in the primary analysis. Model 0 (*n* = 6118) was unadjusted, providing raw associations between *APOE* alleles and biomarkers which may be clinically useful when age‐ and sex‐dependent models are not yet available. Model 1 (*n* = 6118), our primary model, adjusted for age, sex, and study center—demographic covariates known to explain substantial variability in biomarker levels.^14^, ^15^ Model 2 (*n* = 4912) further adjusted for global African and Amerindian ancestry proportions, using European ancestry as the reference, to account for ancestry‐related population structure beyond *APOE*. These model covariates were pre‐selected rather than selected based on statistical significance, which can be driven by sample size rather than biological relevance. Model fit was compared using Akaike information criterion (AIC), calculated for each model among the 4912 participants with complete data on age, sex, study center, and global genetic ancestry; AIC values are provided in Table S2 in supporting information. Across most biomarkers, Model 2 had the lowest AIC, indicating a marginally better fit. However, Model 1 was selected as the primary analytic model to maximize statistical power, given its larger sample size and the consistency of *APOE* effect sizes across models. *APOE* allele effect sizes and *p* values for each model are reported in Table S3 in supporting information. Covariate effect sizes and *p* values for age, sex, study center, and global ancestry from Model 2 are reported in Table S4 in supporting information to provide context for subsequent stratified analyses.

Secondary analyses examined the role of age, sex, genetic analysis group, and genetic ancestry (global and local) in modifying the associations between *APOE* alleles and ATN(I) biomarkers. Given potential non‐linear relationships among age, sex, and biomarker levels, stratified analyses were performed within age (<60, 60–70, >70 years) and sex (male, female) subgroups. Stratification was motivated by well‐established sex differences in AD risk and the late‐onset nature of AD, which may modify *APOE* effects.^14^, ^15^ Biomarker distributions within these subgroups can be found in Figures S3 and S4 in supporting information. Age strata of < 60, 60 to 70, and > 70 years were selected to capture biologically meaningful differences in *APOE*‐related effects while maintaining sufficient sample sizes. Prior studies have shown that ε4 homozygotes begin to show cognitive decline in their 50s, heterozygotes in their 60s, and non‐carriers in their 70s,^48^ supporting the use of these cut‐points to assess age‐dependent associations with AD‐related biomarkers.

Stratified analyses were also performed within the six genetic analysis groups (Central American, Cuban, Dominican, Puerto Rican, Mexican, and South American; *n* = 4893). Effect modification was tested through interaction terms between *APOE* alleles and genetic analysis groups, with heterogeneity tests assessing group differences. To further examine the role of genetic ancestry, we performed additional analyses (*n* = 4912) accounting for both global and local African, European, and Amerindian ancestry proportions. In addition, we tested for effect modification by genetic ancestry by including multiplicative interaction terms between ancestry and *APOE* alleles. Interaction coefficients describe how *APOE* effects change per unit of ancestry but are not directly interpretable at specific ancestry levels. To interpret these interactions, we calculated linear combinations of the main *APOE* effect and the *APOE*–ancestry interaction coefficient at selected ancestry values (0, 0.2, 0.4, 0.6, 0.8, 1.0 for global ancestry; 0, 1, 2 for local ancestry). This provides estimated *APOE* effects at specific ancestry levels, allowing visualization of how *APOE–*ATN(I) biomarker associations differ by ancestry while maintaining stable estimates given the sample size. In each of these secondary analyses, permutation testing was used to assess statistical significance and reduce false positives due to small allele counts, with 10,000 permutations per subgroup.

## 3 RESULTS

### 3.1 Descriptive statistics

**FIGURE 1:**
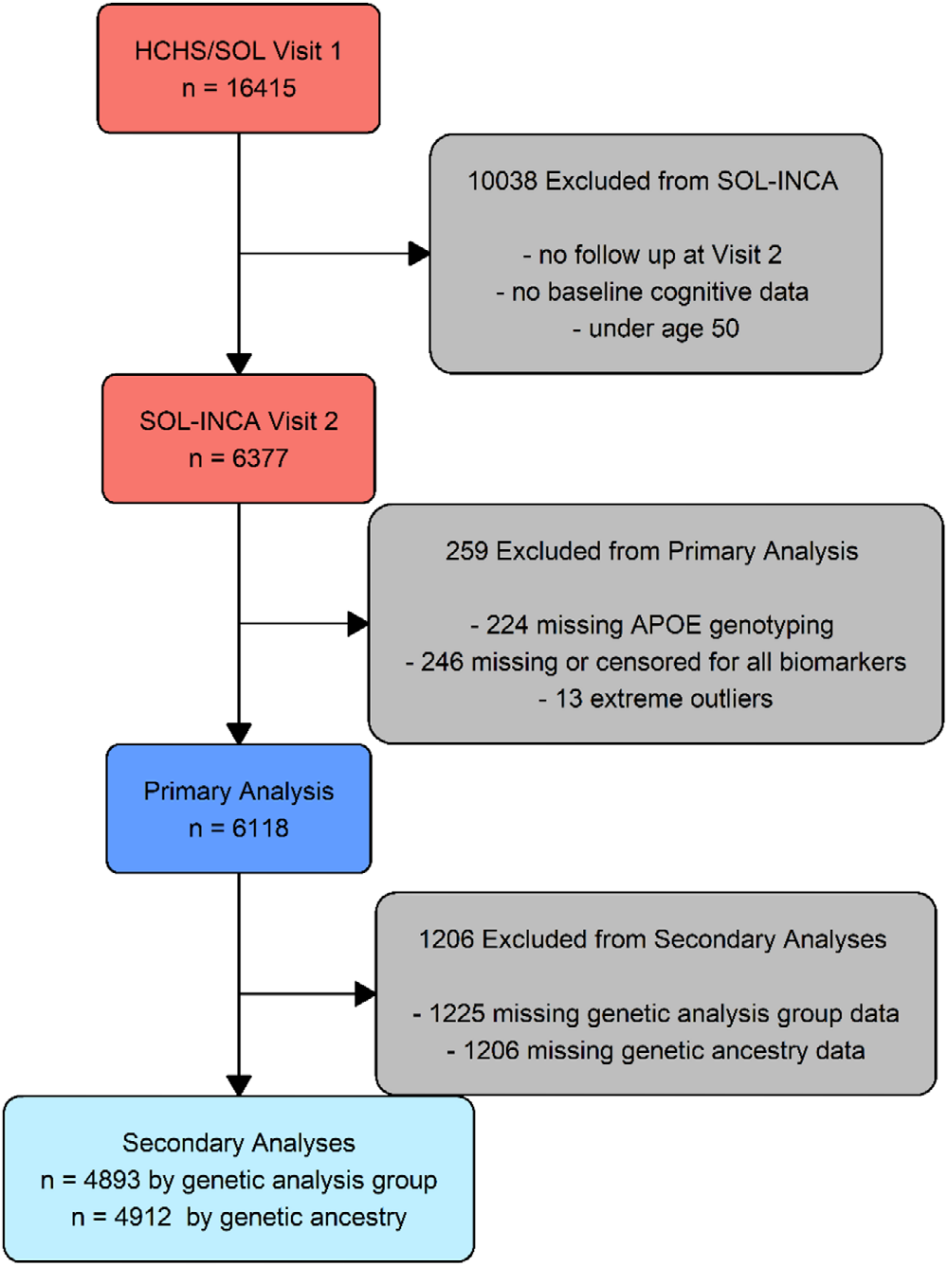
Flowchart of participant selection for primary and secondary analyses. *n* refers to the number of participants. The primary analysis was conducted on 6118 participants, while secondary analyses were performed on 4893 participants with genetic analysis group data and 4912 participants with genetic ancestry data. *APOE*, apolipoprotein E; SOL‐INCA, Study of Latinos–Investigation of Neurocognitive Aging.

A total of 6118 individuals were included in the full analytic sample. Of the original 6377 SOL‐INCA participants, 224 were excluded due to missing *APOE* genotype data, 246 due to missing or censored data for each measured biomarker, and 13 due to extreme outlying biomarker values (Figure 1).

Table 1 summarizes the demographic, genetic, and biomarker characteristics of the analytic sample after batch correction and outlier removal. Results are shown overall as well as stratified by genetic analysis group, which ranged in size from 381 (South American) to 1732 (Mexican). The distribution of sex varied by group, with females comprising 54.4% of the total sample. The average age of participants was 62.05 years (SD = 8.16; range = 49.51–85.82), with the proportion of individuals aged > 70 years highest among Cubans (33.4%) and lowest among Mexicans (17.4%). As expected, the ε3/ε3 genotype was the most common across all groups (70.0% overall), while the ε2/ε2 (< 1% in all groups) and ε4/ε4 (< 3% in all groups) genotypes were rarer. However, *APOE* genotypes and allele frequencies varied by genetic analysis group, with the ε4 allele most frequent among Dominicans (16.4%) and least frequent among South Americans (9.0%), yielding an overall frequency of 11.9%. These genetic analysis group differences in ε4 frequency may reflect variations in ADRD risk that are useful in identifying at‐risk populations. Moreover, global genetic ancestry proportions also differ significantly by genetic analysis group, further highlighting the heterogeneity within Hispanic/Latino populations.

**TABLE 1:** Demographic, genetic, and biomarker characteristics of the SOL‐INCA analytic sample.

Variable	Level	Overall	Central American	Cuban	Dominican	Mexican	Puerto Rican	South American
*N*		6118	482	977	465	1732	856	381
Sex (%)	Female	3931 (54.4)	323 (58.7)	547 (48.0)	321 (58.9)	1130 (55.0)	532 (52.2)	239 (59.9)
	Male	2187 (45.6)	159 (41.3)	430 (52.0)	144 (41.1)	602 (45.0)	324 (47.8)	142 (40.1)
Age (mean [SD])		62.05 (8.16)	61.33 (7.40)	62.63 (8.60)	61.65 (8.23)	61.71 (7.63)	62.97 (8.24)	62.21 (8.08)
Age (min–max)		49.51–85.82	50.00–81.49	50.00–83.01	50.00–85.82	49.51–83.70	49.97–84.81	49.61–82.28
Age (%)	<60	2697 (40.6)	216 (42.2)	392 (32.6)	225 (44.1)	814 (47.3)	335 (36.4)	160 (37.8)
	60−70	2472 (35.4)	213 (39.5)	414 (34.1)	167 (34.8)	670 (35.3)	351 (35.3)	165 (38.4)
	>70	949 (24.0)	53 (18.2)	171 (33.4)	73 (21.2)	248 (17.4)	170 (28.3)	56 (23.7)
***APOE* genetic information**								
*APOE* genotype (%)	ε2/ε2	20 (0.3)	1 (0.0)	4 (0.2)	4 (0.9)	4 (0.2)	3 (0.3)	0 (0.0)
	ε2/ε3	448 (7.4)	32 (6.8)	117 (10.9)	57 (12.6)	84 (3.7)	63 (7.2)	27 (5.4)
	ε2/ε4	66 (1.1)	3 (0.7)	16 (1.2)	16 (4.1)	6 (0.4)	12 (1.0)	2 (0.5)
	ε3/ε3	4304 (70.0)	349 (72.3)	625 (65.4)	256 (56.0)	1307 (76.1)	587 (70.0)	287 (78.8)
	ε3/ε4	1187 (19.6)	89 (18.2)	198 (20.5)	120 (24.1)	305 (18.0)	183 (20.6)	55 (13.0)
	ε4/ε4	93 (1.6)	8 (1.9)	17 (1.8)	12 (2.4)	26 (1.6)	8 (0.8)	10 (2.2)
*APOE* allele (%)	ε2	554 (4.5)	37 (3.8)	141 (6.3)	81 (9.3)	98 (2.2)	81 (4.4)	29 (3.0)
	ε3	10243 (83.5)	819 (84.8)	1565 (81.1)	689 (74.	3003 (87.0)	1420 (83.9)	656 (88.0)
	ε4	1439 (11.9)	108 (11.4)	248 (12.6)	160 (16.4)	363 (10.8)	211 (11.6)	77 (9.0)
**Plasma ATN(I) biomarkers**								
Aβ40 (mean [SD]) (pg/mL)		115.18 (26.03)	114.53 (25.12)	119.95 (28.46)	113.47 (28.26)	113.25 (20.84)	118.02 (29.23)	112.30 (21.83)
Aβ42 (mean [SD]) (pg/mL)		8.04 (1.99)	7.96 (1.96)	8.02 (1.93)	8.18 (1.95)	8.01 (2.13)	8.28 (2.14)	7.86 (2.16)
p‐tau181 (mean [SD]) (pg/mL)		1.76 (0.71)	1.71 (0.68)	1.81 (0.74)	1.77 (0.67)	1.70 (0.69)	1.88 (0.72)	1.75 (0.63)
NfL (mean [SD]) (pg/mL)		16.44 (7.83)	15.48 (7.34)	15.71 (7.87)	15.73 (7.67)	16.69 (7.82)	17.94 (8.27)	15.84 (6.98)
GFAP (mean [SD]) (pg/mL)		146.35 (60.69)	142.96 (59.36)	147.06 (63.83)	160.46 (61.01)	143.10 (56.82)	151.89 (66.11)	140.54 (53.46)
**Global genetic ancestry**								
African (mean [SD])		0.14 (0.18)	0.10 (0.06)	0.15 (0.19)	0.44 (0.16)	0.04 (0.02)	0.22 (0.13)	0.06 (0.07)
European (mean [SD])		0.57 (0.23)	0.46 (0.14)	0.79 (0.20)	0.49 (0.15)	0.48 (0.19)	0.64 (0.12)	0.50 (0.21)
Amerindian (mean [SD])		0.29 (0.23)	0.44 (0.14)	0.06 (0.05)	0.07 (0.02)	0.48 (0.19)	0.14 (0.04)	0.44 (0.21)

*Note*: Mean (SD) and (%) are based on the sampling weights and complex survey design.

Abbreviations: Aβ, amyloid beta; *APOE*, apolipoprotein E; ATN(I), amyloid/tau/neurodegeneration/neuroinflammation; GFAP, glial fibrillary acidic protein; NfL, neurofilament light chain; p‐tau, phosphorylated tau; SD, standard deviation; SOL‐INCA, Study of Latinos–Investigation of Neurocognitive Aging.

### 3.2 Associations between *APOE* alleles and ATN(I) biomarkers

**FIGURE 2:**
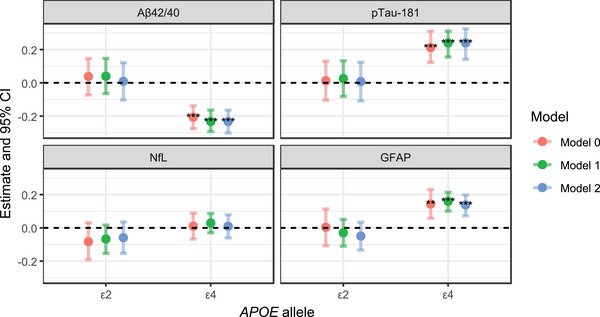
Associations between ATN(I) biomarkers and *APOE* alleles based on the additive inheritance mode. Model 0 (*n* = 6118) was unadjusted; Model 1 (*n* = 6118) was adjusted for age, sex, and study center; Model 2 (*n* = 4912) was adjusted for age, sex, study center, and global genetic ancestry proportions (African and Amerindian, with European as reference). ε3 was used as the reference allele. The *y* axis represents effect estimates for ε2 and ε4 alleles, modeled separately under an additive inheritance mode, where allele counts were coded as 0, 1, or 2. Estimates were standardized by the survey‐weighted standard deviation of each biomarker in the overall cohort and are expressed in SD units. Asterisks indicate statistical significance: **p < 0.01, ***p < 0.001. Aβ, amyloid beta; *APOE*, apolipoprotein E; ATN(I), ATN(I), amyloid/tau/neurodegeneration/neuroinflammation; CI, confidence interval; GFAP, glial fibrillary acidic protein; NfL, neurofilament light chain; p‐tau, phosphorylated tau; SD, standard deviation.

Figure 2 illustrates the associations found between *APOE* alleles and ATN(I) biomarkers for three different additive models, with ε2 and ε4 included together in the same model. The full regression results for each model, including effect estimates, confidence intervals, and *p* values, are provided in Table S3.

Model fit was assessed using AIC in the subset of participants with complete demographic and global ancestry data (*n* = 4912; Table S2). Although Model 2 showed slightly lower AIC values for most biomarkers, effect estimates and statistical significance were highly consistent across Models 0 to 2. Given this consistency and the larger sample size available for Model 1, Model 1 was selected as the primary model for inference. However, for each stratified analysis, global ancestry‐adjusted results are additionally provided in the supporting information.

In Model 1, adjusted for age, sex, and study center, ε4 was associated with lower Aβ42/40 (estimate = –0.233 SD; 95% CI = [–0.293, –0.164]) and higher p‐tau181 (estimate = 0.240 SD; 95% CI = [0.155, 0.311]) and GFAP (estimate = 0.160 SD; 95% CI = [0.100, 0.214]). In contrast, the ε2 allele was not significantly associated with any biomarker, and no significant associations were observed with NfL for either allele.

### 3.3 Stratified analyses by age and sex

**FIGURE 3:**
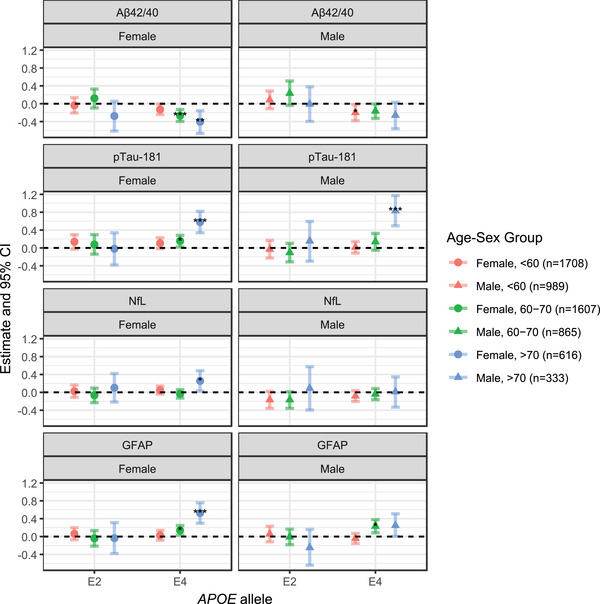
Associations between ATN(I) biomarkers and *APOE* alleles by age and sex. Each biomarker was modeled as a function of *APOE* allele dosage (ε2 or ε4 vs. ε3) within each age–sex subgroup. Models were adjusted for the study center. The estimate represents the standardized change in biomarker level (in SD units) per copy of the specified allele, with ε3 used as the reference. *p* values were estimated based on permutation testing with 10,000 permutations per age‐sex group. Asterisks indicate statistical significance: *p < 0.05, **p < 0.01, ***p < 0.001. Aβ, amyloid beta; *APOE*, apolipoprotein E; ATN(I), ATN(I), amyloid/tau/neurodegeneration/neuroinflammation; CI, confidence interval; GFAP, glial fibrillary acidic protein; NfL, neurofilament light chain; p‐tau, phosphorylated tau; SD, standard deviation.

Using the additive inheritance model adjusted for study center, we next examined ε2 and ε4 together in the same model, stratified by age (< 60, 60–70, and > 70 years) and sex (female and male), to explore potential heterogeneity across age–sex subgroups, regardless of whether the alleles reached significance in the primary analysis (Figure 3 and Table S5 in supporting information). Corresponding results, further adjusting for global genetic ancestry proportions, are shown in Table S6 in supporting information. No significant associations were observed for the ε2 allele. For ε4, key findings include significantly lower Aβ42/40 and higher p‐tau181 and GFAP levels in females > 60 years, while no significant associations were found in females <60 years. Notably, NfL levels were also significantly elevated in females > 70 years. Males <60 years exhibited significantly lower Aβ42/40, while males 60 to 70 years had elevated GFAP, and males > 70 years had elevated p‐tau181 levels. The more consistent signals observed in females may partially reflect the slightly larger female sample size (54.4%).

### 3.4 Stratified analyses by genetic analysis group

**FIGURE 4:**
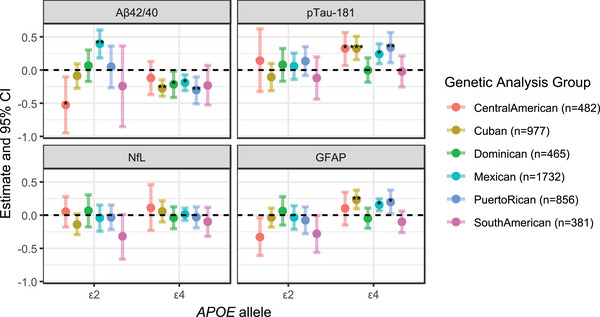
Associations between ATN(I) biomarkers and *APOE* alleles by genetic analysis group. Each biomarker was modeled as a function of *APOE* allele dosage (ε2 or ε4 vs. ε3) within each genetic analysis group. Models were adjusted for age, sex, and study center. The estimate represents the standardized change in biomarker level (in SD units) per copy of the specified allele, with ε3 used as the reference. *p* values were estimated based on permutation testing with 10,000 permutations per genetic analysis group. Tests for heterogeneity across groups were significant for Aβ42/40 (*p* = 0.048), p‐tau181 (*p* = 0.002), and GFAP (*p* = 0.009), but not for NfL. Asterisks indicate statistical significance: *p < 0.05, **p < 0.01, ***p < 0.001. Aβ, amyloid beta; *APOE*, apolipoprotein E; ATN(I), ATN(I), amyloid/tau/neurodegeneration/neuroinflammation; CI, confidence interval; GFAP, glial fibrillary acidic protein; NfL, neurofilament light chain; p‐tau, phosphorylated tau; SD, standard deviation.

Within each genetic analysis group, we estimated associations using the additive inheritance model adjusted for age, sex, and study center, with ε2 and ε4 modeled together (Figure 4 and Table S7 in supporting information). Corresponding global genetic ancestry‐adjusted models are shown in Table S8 in supporting information. Key findings include significant associations between ε2 and Aβ42/40 in Central American (negative) and Mexican (positive) participants, although these opposite directions should be interpreted cautiously, and further work is needed to determine whether this pattern is replicable or influenced by unmeasured factors such as social, health, or lifestyle measures. Additional key findings include significant negative associations between ε4 and Aβ42/40 in Cuban, Dominican, Mexican, and Puerto Rican participants, as well as positive associations with p‐tau181 (Central American, Cuban, Mexican, and Puerto Rican) and GFAP (Cuban, Mexican, and Puerto Rican) across several groups. No significant associations were observed between NfL and either allele.

### 3.5 Analyses by genetic ancestry proportions

The interaction of global genetic ancestry with *APOE* alleles’ effects on biomarkers is presented in Table 2. All interaction analyses used the same age‐, sex‐, and study center‐adjusted additive model, which included main effects for *APOE* and ancestry as well as an *APOE* × ancestry interaction term. ε2 and ε4 were modeled together, and each global ancestry proportion (African, European, Amerindian) was tested in its own model. The only statistically significant interaction was between African ancestry and ε4 on p‐tau181 (estimate = –0.494 SD; 95% CI = [–0.876, –0.110]; *p* value = 0.019). This estimate reflects the additive effect of ε4 alleles, indicating that a full‐scale increase in global African ancestry (from 0% to 100%) is associated with a 0.494 SD decrease in p‐tau181, on average, per ε4 allele. This suggests that African ancestry diminishes the expected ε4‐related increase in p‐tau181 levels.

**TABLE 2:** Associations between ATN(I) biomarkers and *APOE* alleles by global genetic ancestry.

African					
		*APOE* × ancestry interaction		*APOE* main effect	
ATN(I) biomarker	*APOE* allele	Estimate [95% CI]	*p* value	Estimate [95% CI]	*p* value
Aβ42/40	ε2	0.181 [–0.302, 0.672]	0.274	−0.039 [–0.207, 0.129]	0.338
	ε4	0.031 [–0.336, 0.405]	0.447	−0.241 [–0.336, –0.147]	<0.001^***^
p‐tau181	ε2	−0.297 [–0.664, 0.079]	0.159	0.085 [–0.068, 0.240]	0.188
	ε4	−0.494 [–0.876, –0.110]	0.019*	0.325 [0.198, 0.438]	<0.001^***^
NfL	ε2	0.128 [–0.370, 0.626]	0.313	−0.091 [–0.217, 0.033]	0.147
	ε4	−0.255 [–0.600, 0.103]	0.136	0.055 [–0.036, 0.140]	0.192
GFAP	ε2	0.068 [–0.247, 0.379]	0.396	−0.061 [–0.181, 0.056]	0.241
	ε4	−0.142 [–0.577, 0.297]	0.271	0.165 [0.086, 0.247]	0.005^**^

European					
		*APOE* × ancestry interaction		*APOE* main effect	
ATN(I) biomarker	*APOE* allele	Estimate [95% CI]	*p* value	Estimate [95% CI]	*p* value
Aβ42/40	ε2	−0.293 [–0.810, 0.224]	0.166	0.216 [–0.112, 0.534]	0.130
	ε4	−0.138 [–0.466, 0.190]	0.293	−0.138 [–0.345, 0.060]	0.178
p‐tau181	ε2	0.013 [–0.424, 0.452]	0.476	0.016 [–0.240, 0.282]	0.464
	ε4	0.381 [–0.008, 0.763]	0.059	0.024 [–0.198, 0.240]	0.427
NfL	ε2	−0.128 [–0.562, 0.307]	0.317	0.022 [–0.281, 0.319]	0.438
	ε4	0.230 [–0.103, 0.549]	0.160	−0.120 [–0.307, 0.064]	0.201
GFAP	ε2	−0.063 [–0.395, 0.280]	0.416	0.010 [–0.198, 0.214]	0.477
	ε4	0.102 [–0.214, 0.428]	0.325	0.089 [–0.119, 0.297]	0.262

Amerindian					
		*APOE* × ancestry interaction		*APOE* main effect	
ATN(I) biomarker	*APOE* allele	Estimate [95% CI]	*p* value	Estimate [95% CI]	*p* value
Aβ42/40	ε2	−0.081 [–0.802, 0.638]	0.402	0.048 [–0.121, 0.216]	0.297
	ε4	0.016 [–0.293, 0.328]	0.463	−0.224 [–0.336, –0.121]	0.002^**^
p‐tau181	ε2	0.212 [–0.325, 0.734]	0.264	−0.035 [–0.198, 0.121]	0.358
	ε4	−0.021 [–0.367, 0.325]	0.472	0.240 [0.105, 0.367]	<0.001^***^
NfL	ε2	−0.073 [–0.562, 0.421]	0.417	−0.050 [–0.179, 0.079]	0.292
	ε4	−0.020 [–0.307, 0.268]	0.473	0.014 [–0.100, 0.126]	0.426
GFAP	ε2	−0.313 [–0.791, 0.145]	0.171	0.007 [–0.115, 0.129]	0.467
	ε4	−0.054 [–0.346, 0.247]	0.415	0.153 [0.043, 0.264]	0.014^*^

*Note*: Each biomarker was modeled as a function of *APOE* allele dosage (ε2 or ε4 vs. ε3), global genetic ancestry proportion (African, European, or Amerindian), and their interaction. Models were adjusted for age, sex, and study center. The *APOE* × ancestry interaction estimate represents the estimated change in biomarker level per copy of the specified *APOE* allele, per full‐scale (0 to 1) increase in ancestry proportion. The *APOE* main effect estimate represents the estimated change in biomarker level per copy of the specified *APOE* allele, in the absence of the specified ancestry. Estimates were standardized by the survey‐weighted standard deviation of each biomarker in the overall cohort and are expressed in SD units. *p* values were estimated based on permutation testing with 10,000 permutations. Asterisks indicate statistical significance.

Abbreviations: Aβ, amyloid beta; *APOE*, apolipoprotein E; ATN(I), amyloid/tau/neurodegeneration/neuroinflammation; CI, confidence interval; GFAP, glial fibrillary acidic protein; NfL, neurofilament light chain; p‐tau, phosphorylated tau; SD, standard deviation.

* *p* < 0.05.

** *p* < 0.01.

*** *p* < 0.001.

**FIGURE 5:**
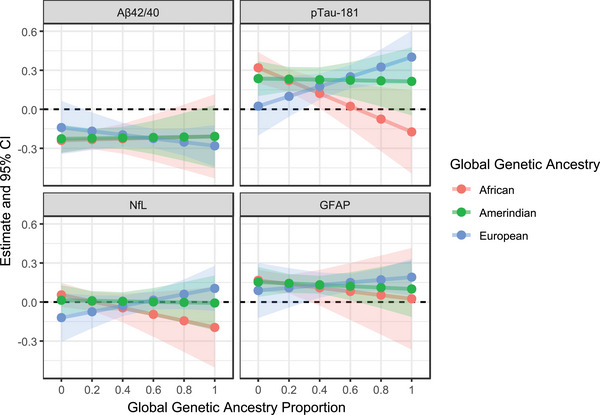
Interaction of global genetic ancestry proportion and ε4 on ATN(I) biomarkers. Associations were evaluated at ancestry proportions of 0, 0.2, 0.4, 0.8, and 1. Effect estimates are shown by points, with 95% confidence intervals represented by shaded regions. Estimates were derived from linear combinations of regression coefficients, adjusting for age, sex, and study center, and were standardized to the survey‐weighted SD of each biomarker in the overall cohort. Aβ, amyloid beta; ATN(I), ATN(I), amyloid/tau/neurodegeneration/neuroinflammation; CI, confidence interval; GFAP, glial fibrillary acidic protein; NfL, neurofilament light chain; p‐tau, phosphorylated tau; SD, standard deviation.

This pattern is demonstrated in Figure 5, which illustrates the estimates derived from linear combinations of regression coefficients. At lower African ancestry proportions, ε4 was significantly associated with higher p‐tau181 levels, in addition to higher GFAP and lower Aβ42/40, while these associations were attenuated at higher African ancestry proportions. The weaker associations at higher African ancestry proportions may offset the stronger associations at lower levels, resulting in non‐significant interaction terms overall for both Aβ42/40 and GFAP. The opposite trend was observed for European ancestry, for which point estimates suggested a greater magnitude of ε4 effects at higher European ancestry proportions for Aβ42/40, p‐tau181, and GFAP, and smaller magnitudes at lower proportions. There was no clear pattern of change in ε4 associations across global Amerindian ancestry proportions. Similarly, no clear patterns were observed for the ε2 allele across any global ancestry proportion (Table S9 and Figure S5 in supporting information).

**FIGURE 6:**
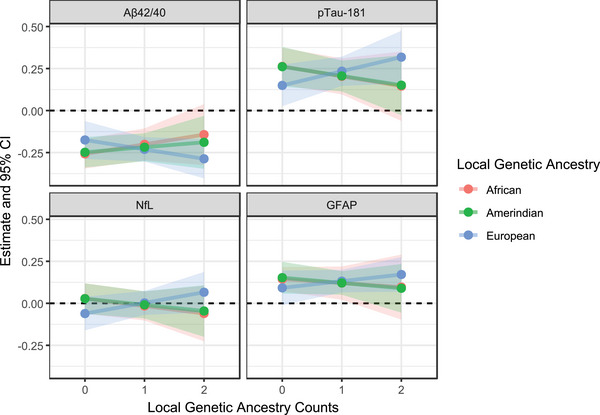
Interaction of local genetic ancestry counts and ε4 on ATN(I) biomarkers. Associations were evaluated at ancestry counts of 0, 1, and 2. Effect estimates are shown by points, with 95% confidence intervals represented by shaded regions. Estimates were derived from linear combinations of regression coefficients, adjusting for age, sex, study center, and global genetic ancestry proportions (African and Amerindian, with European as reference), and were standardized to the survey‐weighted SD of each biomarker in the overall cohort. Aβ, amyloid beta; ATN(I), ATN(I), amyloid/tau/neurodegeneration/neuroinflammation; CI, confidence interval; GFAP, glial fibrillary acidic protein; NfL, neurofilament light chain; p‐tau, phosphorylated tau; SD, standard deviation.

For local ancestry, we applied the same age‐, sex‐, and study center‐adjusted additive model, additionally adjusting for global ancestry. The model included *APOE* and local ancestry main effects plus an *APOE* × local ancestry interaction term, with ε2 and ε4 evaluated together and each local ancestry count assessed independently. The associations between ATN(I) biomarkers and *APOE* alleles by local genetic ancestry, including interaction term and *APOE* term estimates and *p* values, are summarized in Table S10 in supporting information. No significant interactions were found between local ancestry and *APOE* alleles on any ATN(I) biomarker. Local ancestry counts were broadly distributed in the cohort (24% with ≥1 African‐ancestry copy, 78% with ≥1 European‐ancestry copy, and 44% with ≥1 Amerindian‐ancestry copy), suggesting the lack of significant interactions is unlikely to reflect limited variation. Figures 6 and S6 in supporting information illustrate these interactions at different local ancestry counts, with numerical estimates summarized in Table S11 in supporting information. Overall, the *APOE*–biomarker associations across African and European local ancestry counts mirrored the global ancestry patterns. For Amerindian ancestry, in which global analyses showed no clear pattern of change, local ancestry results suggested modest shifts in ε4 effects, in which Aβ42/40 levels increased, and p‐tau181, GFAP, and NfL levels decreased as Amerindian counts rose from 0 to 2.

## 4 DISCUSSION

This study investigated the associations of *APOE* alleles and ATN(I) biomarkers (Aβ42/40, p‐tau181, NfL, and GFAP) in a diverse Hispanic/Latino population, with secondary analyses accounting for six genetic analysis groups (Central American, Cuban, Dominican, Mexican, Puerto Rican, and South American) and three genetic ancestries (African, Amerindian, and European), as well as age and sex stratifications. Stratified analyses showed differences in *APOE*–ATN(I) associations across genetic analysis groups and suggested ancestry‐related effect modification, especially with p‐tau181. While results from stratified analyses are limited due to modest sample sizes and low allele frequencies in some subgroups, they suggest that AD screening efforts relying on plasma ATN(I) biomarkers may need to consider individual genetic patterns, potentially accounting for admixture.

Consistent with previous research,^5^, ^23^, ^49^, ^50^ our findings confirm that ε4 is associated with Aβ42/40, p‐tau181, and GFAP biomarker levels in US Hispanics/Latinos. For Aβ42/40, the standardized effect size in our sample (–0.233 SD) is comparable to prior reports in European ancestry cohorts, in which ε4 was significantly associated with lower plasma Aβ42/40 (effect size = –0.212 SD; 95% CI = [–0.257, –0.121]; *p* value = 6.46 × 10^−20^).^49^ For p‐tau181 and GFAP, ε4 was significantly associated with moderately higher levels, highlighting potential effects on tau pathology and neuroinflammation. Importantly, these results extend prior work by exhibiting these associations in a younger, population‐based cohort of midlife Latino adults, which may have implications for early risk detection. In contrast, the ε2 allele was not significantly associated with biomarker levels in the overall sample, though stratified analyses suggested potential protective effects in select genetic analysis groups. The lack of significant ε2 associations in the full analytic sample may, however, reflect its low frequency or the limited statistical power to detect modest associations. Consistent with some studies^5^, ^50^ but contrary to others,^4^, ^51^, ^52^ the primary analysis found no significant associations with NfL, suggesting that *APOE* effects on neurodegeneration may depend on cohort characteristics. NfL is a general marker of axonal damage rather than a specific indicator of AD,^7^ and its levels typically increase later in the disease course.^53^ Given that our study population is largely younger and cognitively unimpaired, widespread neurodegeneration is not expected and may not yet be detectable. Studies reporting significant *APOE*–NfL associations^4^, ^51^, ^52^ generally included older or clinically diagnosed AD participants, in whom neurodegeneration is more pronounced. Alternatively, *APOE* may act as an effect modifier in the relationship between AD pathology and neurodegeneration, or null findings may reflect unmeasured confounding, such as kidney function, which influences NfL clearance.^54^, ^55^

While our primary findings showed consistent associations between ε4 and lower Aβ42/40 and higher p‐tau181 and GFAP levels, secondary analyses revealed variability in the magnitude of these associations across genetic analysis groups and ancestry proportions. Given modest sample sizes (< 1000) in some subgroups and moderate ε4 allele frequencies (≈ 12%), these differences should be interpreted cautiously and require replication in larger cohorts. Among ε4 carriers, decreased Aβ42/40 and increased p‐tau181 and GFAP levels were most pronounced in Cuban and Puerto Rican analysis groups, which had the highest European ancestry proportions (> 50%), and in the Mexican analysis group, which had the lowest African ancestry proportion (< 5%). These findings align with external studies reporting similarly elevated plasma p‐tau181 levels among ε4 carriers in Caribbean Hispanic (Washington Heights and Inwood Community Aging Project: estimate = 0.31 SD; 95% CI = [0.06, 0.55]) and Mexican American (Health & Aging Brain Study–Health Disparities: estimate = 0.32 SD; 95% CI = [0.11, 0.53]) cohorts,^23^ which fall within the general range of the ε4–p‐tau181 effects observed in our Cuban (0.33 [0.16, 0.51] SD), Puerto Rican (0.34 [0.11, 0.57] SD), and Mexican (0.24 [0.10, 0.40] SD) groups, though differences in sample size, population structure, and ancestry composition may contribute to variation across studies. These patterns are consistent with evidence that ADRD risk varies by genetic ancestry, in which individuals with African ancestry tend to show lower risk than those with European ancestry,^56^, ^57^ potentially explaining why non‐Hispanic Black individuals also exhibit weaker *APOE*–ADRD associations compared to non‐Hispanic White individuals.^21^, ^58^

To further investigate the role of ancestry, we explored the impact of local versus global ancestry on *APOE* allele associations with ATN(I) biomarkers. Patterns for local genetic ancestry largely mirrored those for global ancestry, and no statistically significant interactions were observed. This suggests that *APOE* ’s interaction with ancestry‐related factors outside the *APOE* region, rather than near the *APOE* locus itself, may be modifying *APOE*’s effects on ATN(I) biomarker levels. These modifiers could reflect environmental or socioeconomic exposures correlated with ancestry, or ancestry‐specific patterns of linkage disequilibrium elsewhere in the genome that may interact with *APOE* to influence biomarker levels. Two prior Caribbean Hispanic studies^59^, ^60^ reported protective effects of local African ancestry on ε4‐related AD risk, which contrasts with our null findings. However, these results are not directly comparable to ours, as those studies analyzed AD outcomes rather than biomarker variation and were conducted in populations with potentially different genetic backgrounds and *APOE*–region variation, which may contribute to differences in observed effects. Larger studies will be needed to more fully assess whether local ancestry influences *APOE*–biomarker relationships.

To address whether ancestry‐related heterogeneity might be driven by differences in age distributions or population substructure, we conducted supplementary stratified analyses by age (Figure S7 and Table S12 in supporting information) and genetic analysis group (Figure S8 and Table S13 in supporting information). Across these analyses, we observed variation in ε4 × global genetic ancestry interactions by both age and genetic analysis group, though many effect sizes were imprecise with wide confidence intervals. Because each stratum combines different mixtures of age, sex, and ancestry, some of this variability may arise from compositional differences rather than true effect modification. This reinforces the need for larger datasets that can model multiple stratification variables to isolate ancestry‐specific *APOE* effects.

This study is the first to investigate genetic variation in relation to AD biomarker levels in Hispanic/Latino populations while incorporating genetic background and ancestry. A key strength of this study is its diverse Hispanic/Latino population, allowing for the exploration of effects across six genetic analysis groups and three genetic ancestries. Another strength is its use of complex survey methods to account for stratification, clustering, and probability weighting in HCHS/SOL, which allows for generalizations to the target population. Additionally, adjustment for plate‐induced batch effects and extreme outliers helps ensure the robustness of biomarker measurements.

Despite these strengths, there are still several limitations to consider. Based on previous studies,^7^, ^53^ we assumed that blood‐based ATN(I) biomarkers accurately reflect the pathological processes occurring in the brain and are not significantly influenced by peripheral factors unrelated to ADRD. Additionally, the low frequencies of rare genotypes such as ε2/ε2 and ε4/ε4 forced the analysis of *APOE* alleles over genotypes. Even still, the small sample sizes of the rarer ε2 and ε4 alleles led to large confidence intervals that may have limited statistical power to detect significant associations, especially in stratified analyses. We also did not account for the ε2/ε4 genotype, which may have opposing effects that could bias results toward the null.^21^, ^50^ While a diverse study population is a key strength, the smaller sample sizes for some genetic analysis groups, such as South Americans, Central Americans, and Cubans, may have also reduced statistical power. Importantly, our interpretations were based on the presumption of homogeneity within genetic analysis groups, in which we assumed that Hispanic/Latino background groups share sufficiently similar ancestral, genotypic, and environmental characteristics to make meaningful comparisons. Differences in the distributions of covariates that may modify *APOE*–biomarker associations limit conclusions about whether observed between‐group differences are driven by genetic factors alone. These findings should therefore be interpreted as exploratory and hypothesis generating. Another key limitation is the limited ability to directly compare effect sizes to prior studies. Published *APOE*–biomarker associations differ widely in study design (clinical ADRD vs. population based), biomarker measurement modality (plasma, CSF, PET), analytic approaches (transformations, covariates), and underlying population ancestry. These differences complicate direct numerical comparison, but broader patterns can still be evaluated. To place our results within this context, we compiled a summary table (Table S14 in supporting information) outlining reported *APOE–*biomarker effect sizes across prior studies^4^, ^5^, ^23^, ^49^, ^50^, ^51^, ^52^, ^61^, ^62^, ^63^, ^64^, ^65^, ^66^ while highlighting relevant design differences. Collectively, these comparisons suggest that study design, biomarker measurement, and population ancestry may contribute to the varying *APOE* effect sizes across the literature.

In summary, this study supports the importance of including genetic ancestry in studies of ADRD, especially in populations at high risk. Future research should examine factors such as education, health‐care access, diet, exercise, smoking status, and air pollution that may interact with *APOE* alleles. Models could also incorporate cardiometabolic conditions such as hypertension and diabetes, which are linked to ADRD risk^15^, ^67^ but show unclear associations with ATN(I) biomarkers,^68^, ^69^, ^70^ to explore their potential mediating role between genetics and biomarker levels. Additionally, longitudinal studies could assess whether the observed ancestry‐ and background‐specific differences translate to differences in disease progression and clinical outcomes. Finally, studies incorporating multi‐omics approaches, including transcriptomics and proteomics,^71^, ^72^ could help explain mechanisms underlying ancestry‐specific *APOE* effects or other pathways contributing to ADRD risk.

## CONFLICT OF INTEREST STATEMENT

Dr. Alberto R. Ramos reports receiving grant support from Axsome Pharmaceuticals, consulting fees from Jazz Pharmaceuticals, honoraria from the Medical Education Speakers Network, and participation in the National Institutes of Health/National Heart, Lung and Blood Institute's Sleep Disorders Research Advisory Board. Dr. Hector M. Gonzalez reports receiving consulting fees from UTSAHSC ADRC, receiving REMIND honorarium from UCI, and receiving support from the National Academy of Science, Engineering & Medicine for attending a meeting. Dr. Paola Filigrana reports receiving grant support from the Alzheimer's Association (24AARFD‐1242387). Dr. Krista M. Perreira reports receiving grant support from the NIH, with payments made to UNC Chapel Hill. Dr. Wassim Tarraf reports having the role of a section editor for the ADJ. Dr. Tatjana Rundek reports receiving NIH grant support, with payments made to U of Miami. All other authors report no conflicts of interest.

## CONSENT STATEMENT

The HCHS/SOL was approved by the institutional review boards (IRBs) at each field center, where all participants gave written informed consent, and by the Non‐Biomedical IRB at the University of North Carolina at Chapel Hill, to the HCHS/SOL Data Coordinating Center. All IRBs approving the HCHS/SOL study are: Non‐Biomedical IRB at the University of North Carolina at Chapel Hill, Chapel Hill, NC; Einstein IRB at the Albert Einstein College of Medicine of Yeshiva University, Bronx, NY; IRB at Office for the Protection of Research Subjects (OPRS), University of Illinois at Chicago, Chicago, IL; Human Subject Research Office, University of Miami, Miami, FL; Institutional Review Board of San Diego State University, San Diego, CA. All methods and analyses of HCHS/SOL participants’ materials and data were carried out in accordance with human subject research guidelines and regulations. This work was approved by the Beth Israel Deaconess Medical Center Committee on Clinical Investigations.

## Supporting information



Supplementary table S1. Coefficients of variation (CV%) for plasma ATN(I) biomarkers.
Supplementary figure 1. Heatmap of unadjusted pairwise Spearman correlation coefficients between plasma ATN(I) biomarkers.
Supplementary figure 2. Distributions of plasma ATN(I) biomarkers and model residuals.
Supplementary table S2. Model fit comparison across nested primary analysis regression models using AIC.
Supplementary table S3. Associations between ATN(I) biomarkers and *APOE* alleles based on additive inheritance mode.
Supplementary table S4. Covariate associations with plasma ATN(I) biomarkers in primary analysis.
Supplementary figure 3. Distributions of plasma ATN(I) biomarkers by age.
Supplementary figure 4. Distribution of plasma ATN(I) biomarkers by sex.
Supplementary table S5. Associations between ATN(I) biomarkers and *APOE* allele (additive mode) by age and sex.
Supplementary table S6. Associations between ATN(I) biomarkers and *APOE* allele (additive mode) by age and sex, adjusted for global ancestry.
Supplementary table S7. Associations between ATN(I) biomarkers and *APOE* allele (additive mode) by genetic analysis group.
Supplementary table S8. Associations between ATN(I) biomarkers and *APOE* allele (additive mode) by genetic analysis group, adjusted for global ancestry.
Supplementary table S9. Interaction of global genetic ancestry proportion and *APOE* alleles (additive) on ATN(I) biomarkers.
Supplementary figure 5. Interaction of global genetic ancestry proportion and *APOE* ε2 allele on ATN(I) biomarkers.
Supplementary table S10. Associations between ATN(I) biomarkers and *APOE* allele by local genetic ancestry.
Supplementary figure 6. Interaction of local genetic ancestry counts and *APOE* ε2 allele on ATN(I) biomarkers.
Supplementary table S11. Stratified interaction of local ancestry proportion and *APOE* alleles on ATN(I) biomarkers.
Supplementary figure 7. Associations between ATN(I) biomarkers and APOE allele by global genetic ancestry, stratified by age.
Supplementary table S12. Associations between ATN(I) biomarkers and *APOE* allele by global genetic ancestry, stratified by age.
Supplementary figure 8. Associations between ATN(I) biomarkers and *APOE* allele by global genetic ancestry, stratified by genetic analysis group.
Supplementary table S13. Associations between ATN(I) biomarkers and *APOE* allele by global genetic ancestry, stratified by genetic analysis group.
Supplementary table S14. Comparisons of design and results of prior *APOE* and AD biomarker studies.

Supporting information

## References

[1] O'Bryant SE , Zhang F , Petersen M , et al. Neurodegeneration from the AT(N) framework is different among Mexican Americans compared to non‐Hispanic Whites: a Health & Aging Brain among Latino Elders (HABLE) Study. Alzheimers Dement Diagn Assess Dis Monit. 2022;14(1):e12267. 10.1002/dad2.12267 PMC8828994 35155729

[2] Gleason CE , Zuelsdorff M , Gooding DC , et al. Alzheimer's disease biomarkers in Black and non‐Hispanic White cohorts: a contextualized review of the evidence. Alzheimers Dement. 2022;18(8):1545‐1564. 34870885 10.1002/alz.12511 PMC9543531

[3] Scheltens P , Strooper BD , Kivipelto M , et al. Alzheimer's disease. Lancet. 2021;397(10284):1577‐1590. 33667416 10.1016/S0140-6736(20)32205-4 PMC8354300

[4] Ng TKS , Beck T , Boyle P , et al. *APOE4*, blood neurodegenerative biomarkers, and cognitive decline in community‐dwelling older adults. JAMA Netw Open. 2025;8(5):e258903. 40332937 10.1001/jamanetworkopen.2025.8903 PMC12059971

[5] Chatterjee P , Pedrini S , Doecke JD , et al. Plasma Aβ42/40 ratio, p‐tau181, GFAP, and NfL across the Alzheimer's disease continuum: a cross‐sectional and longitudinal study in the AIBL cohort. Alzheimers Dement. 2023;19(4):1117‐1134. 36574591 10.1002/alz.12724

[6] Imbimbo BP , Watling M , Imbimbo C , Nisticò R . Plasma ATN(I) classification and precision pharmacology in Alzheimer's disease. Alzheimers Dement. 2023;19(10):4729‐4734. 37079778 10.1002/alz.13084

[7] Leuzy A , Mattsson‐Carlgren N , Palmqvist S , Janelidze S , Dage JL , Hansson O . Blood‐based biomarkers for Alzheimer's disease. EMBO Mol Med. 2022;14(1):e14408. 34859598 10.15252/emmm.202114408 PMC8749476

[8] Benedet AL , Milà‐Alomà M , Vrillon A , et al. Differences between plasma and cerebrospinal fluid glial fibrillary acidic protein levels across the Alzheimer disease continuum. JAMA Neurol. 2021;78(12):1471‐1483. 34661615 10.1001/jamaneurol.2021.3671 PMC8524356

[9] Cai H , Pang Y , Fu X , Ren Z , Jia L . Plasma biomarkers predict Alzheimer's disease before clinical onset in Chinese cohorts. Nat Commun. 2023;14:6747. 37875471 10.1038/s41467-023-42596-6 PMC10597998

[10] Dresse MT , Ferreira PCL , Prasadan A , et al. Plasma biomarkers identify brain ATN abnormalities in a dementia‐free population‐based cohort. Alzheimers Res Ther. 2025;17(1):173. 40713737 10.1186/s13195-025-01803-w PMC12291478

[11] Rauchmann BS , Schneider‐Axmann T , Perneczky R . Associations of longitudinal plasma p‐tau181 and NfL with tau‐PET, Aβ‐PET and cognition. J Neurol Neurosurg Psychiatry. 2021;92(12):1289‐1295. 34187867 10.1136/jnnp-2020-325537 PMC8606440

[12] Sun Q , Ni J , Wei M , et al. Plasma β‐amyloid, tau, neurodegeneration biomarkers and inflammatory factors of probable Alzheimer's disease dementia in Chinese individuals. Front Aging Neurosci. 2022;14:963845. 36062146 10.3389/fnagi.2022.963845 PMC9433929

[13] Cullen NC , Leuzy A , Janelidze S , et al. Plasma biomarkers of Alzheimer's disease improve prediction of cognitive decline in cognitively unimpaired elderly populations. Nat Commun. 2021;12:3555. 34117234 10.1038/s41467-021-23746-0 PMC8196018

[14] Safiri S , Ghaffari Jolfayi A , Fazlollahi A , et al. Alzheimer's disease: a comprehensive review of epidemiology, risk factors, symptoms diagnosis, management, caregiving, advanced treatments and associated challenges. Front Med. 2024;11:1474043. https://www.frontiersin.org/journals/medicine/articles/10.3389/fmed.2024.1474043/full 10.3389/fmed.2024.1474043 PMC11682909 39736972

[15] Aiello AE , Momkus J , Stebbins RC , et al. Risk factors for Alzheimer's disease and cognitive function before middle age in a U.S. representative population‐based study. Lancet Reg Health Am. 2025;45:101087. 40242320 10.1016/j.lana.2025.101087 PMC12001091

[16] Lumsden AL , Mulugeta A , Zhou A , Hyppönen E . Apolipoprotein E (APOE) genotype‐associated disease risks: a phenome‐wide, registry‐based, case‐control study utilising the UK Biobank. EBioMedicine. 2020;59:102954. 32818802 10.1016/j.ebiom.2020.102954 PMC7452404

[17] Mares J , Kumar G , Sharma A , et al. APOE ε4–associated heterogeneity of neuroimaging biomarkers across the Alzheimer's disease continuum. Alzheimers Dement. 2025;21(1):e14392. 39575672 10.1002/alz.14392 PMC11775459

[18] jr Jack . CR , Andrews JS , Beach TG , et al. Revised criteria for diagnosis and staging of Alzheimer's disease: Alzheimer's Association Workgroup. Alzheimers Dement. 2024;20(8):5143‐5169. 38934362 10.1002/alz.13859 PMC11350039

[19] Howell JC , Watts KD , Parker MW , et al. Race modifies the relationship between cognition and Alzheimer's disease cerebrospinal fluid biomarkers. Alzheimers Res Ther. 2017;9:88. 29096697 10.1186/s13195-017-0315-1 PMC5668981

[20] Lim AC , Barnes LL , Weissberger GH , et al. Quantification of race/ethnicity representation in Alzheimer's disease neuroimaging research in the USA: a systematic review. Commun Med. 2023;3(1):1‐12. 37491471 10.1038/s43856-023-00333-6 PMC10368705

[21] Belloy ME , Andrews SJ , Le Guen Y , et al. *APOE* genotype and Alzheimer disease risk across age, sex, and population ancestry. JAMA Neurol. 2023;80(12):1284. 37930705 10.1001/jamaneurol.2023.3599 PMC10628838

[22] Huggins LK , Min SH , Kaplan S , Wei J , Welsh‐Bohmer K , Xu H . Meta‐analysis of variations in association between APOE ε4 and Alzheimer's disease and related dementias across Hispanic regions of origin. J Alzheimers Dis JAD. 2023;93(3):1095‐1109. 37182874 10.3233/JAD-221167 PMC10441171

[23] Barral S , Yang Z , Phillips N , et al. APOE and Alzheimer's disease and related dementias risk among 12,221 Hispanics/Latinos. Alzheimers Dement. 2025;21(4):e70138. 40219824 10.1002/alz.70138 PMC11992591

[24] Xiao C , Pappas I , Aksman LM , O'Bryant SE , Toga AW , the Health and Aging Brain Study (HABS‐HD) Study Team , Alzheimer's Disease Neuroimaging Initiative . Comparison of genetic and health risk factors for mild cognitive impairment and Alzheimer's disease between Hispanic and non‐Hispanic white participants. Alzheimers Dement. 2023;19(11):5086‐5094. 37104247 10.1002/alz.13110 PMC13390812

[25] O'Bryant SE , Petersen M , Hall J , Johnson LA , Team for the HHS . Medical comorbidities and ethnicity impact plasma Alzheimer's disease biomarkers: important considerations for clinical trials and practice. Alzheimers Dement. 2023;19(1):36‐43. 35235702 10.1002/alz.12647 PMC13270989

[26] Royse SK , Cohen AD , Snitz BE , Rosano C . Differences in Alzheimer's disease and related dementias pathology among African American and Hispanic women: a qualitative literature review of biomarker studies. Front Syst Neurosci. 2021;15:685957. https://www.frontiersin.org/journals/systems‐neuroscience/articles/10.3389/fnsys.2021.685957/full 34366799 10.3389/fnsys.2021.685957 PMC8334184

[27] Petersen ME , Zhang F , Hall J , et al. Characterization of plasma AT(N) biomarkers among a racial and ethnically diverse community‐based cohort: an HABS‐HD study. Alzheimers Dement Transl Res Clin Interv. 2025;11(1):e70045. 10.1002/trc2.70045 PMC11837735 39975470

[28] Granot‐Hershkovitz E , Tarraf W , Kurniansyah N , et al. APOE alleles’ association with cognitive function differs across Hispanic/Latino groups and genetic ancestry in the study of Latinos‐investigation of neurocognitive aging (HCHS/SOL). Alzheimers Dement. 2021;17(3):466‐474. 33155766 10.1002/alz.12205 PMC8016734

[29] Gillespie NA , Elman JA , McKenzie RE , et al. The heritability of blood‐based biomarkers related to risk of Alzheimer's disease in a population‐based sample of early old‐age men. Alzheimer's Dement. 2024;20:356‐365. 10.1002/alz.13407 37622539 PMC10843753

[30] Miller MW , Wolf EJ , Zhao X , Logue MW , Hawn SE . An EWAS of dementia biomarkers and their associations with age, African ancestry, and PTSD. Clin Epigenetics. 2024;16:38. 38431614 10.1186/s13148-024-01649-3 PMC10908031

[31] LaVange LM , Kalsbeek W , Sorlie PD , et al. Sample design and cohort selection in the Hispanic Community Health Study/Study of Latinos. Ann Epidemiol. 2010;20(8):642‐649. 20609344 10.1016/j.annepidem.2010.05.006 PMC2921622

[32] González HM , Tarraf W , Fornage M , et al. A research framework for cognitive aging and Alzheimer's disease among diverse US Latinos: design and implementation of the Hispanic Community Health Study/Study of Latinos – Investigation of Neurocognitive Aging (SOL‐INCA). Alzheimers Dement J Alzheimers Assoc. 2019;15(12):1624‐1632. 10.1016/j.jalz.2019.08.192 PMC6925624 31759880

[33] irzada A , Cai J , Heiss G , et al. Evolving Science on Cardiovascular Disease Among Hispanic/Latino Adults: JACC International. J Am Coll Cardiol. 2023;81(15):1505‐1520. 37045521 10.1016/j.jacc.2023.02.023 PMC12330305

[34] Anita NZ , Tarraf W , Kuwayama S , et al. Kidney function is associated with plasma ATN biomarkers among Hispanics/Latinos: SOL‐INCA and HCHS/SOL results. Alzheimers Res Ther. 2025;17(1):137. 40537838 10.1186/s13195-025-01786-8 PMC12180232

[35] Hansson O , Lehmann S , Otto M , Zetterberg H , Lewczuk P . Advantages and disadvantages of the use of the CSF amyloid β (Aβ) 42/40 ratio in the diagnosis of Alzheimer's disease. Alzheimers Res Ther. 2019;11:34. 31010420 10.1186/s13195-019-0485-0 PMC6477717

[36] Vergallo A , Mégret L , Lista S , et al. Plasma amyloid β 40/42 ratio predicts cerebral amyloidosis in cognitively normal individuals at risk for Alzheimer's disease. Alzheimers Dement. 2019;15(6):764‐775. 31113759 10.1016/j.jalz.2019.03.009

[37] Fornage M , Tarraf W , Daviglus ML , et al. Association of epigenetic aging with plasma biomarkers of amyloid, tau, neurodegeneration, and neuroinflammation in Hispanic/Latino adults. Clin Epigenetics. 2025;17(1):136. 40750903 10.1186/s13148-025-01941-w PMC12315259

[38] Sofer T , Wong Q , Hartwig FP , et al. Genome‐wide association study of blood pressure traits by Hispanic/Latino background: the Hispanic Community Health Study/Study of Latinos. Sci Rep. 2017;7:10348. 28871152 10.1038/s41598-017-09019-1 PMC5583292

[39] Conomos MP , Laurie CA , Stilp AM , et al. Genetic diversity and association studies in US Hispanic/Latino populations: applications in the Hispanic Community Health Study/Study of Latinos. Am J Hum Genet. 2016;98(1):165‐184. 26748518 10.1016/j.ajhg.2015.12.001 PMC4716704

[40] Rao H , Weiss MC , Moon JY , et al. Advancements in genetic research by the Hispanic Community Health Study/Study of Latinos: a 10‐year retrospective review. Hum Genet Genomics Adv. 2024;6(1):100376. 10.1016/j.xhgg.2024.100376 PMC11754138 39473183

[41] González HM , Tarraf W , Jian X , et al. Apolipoprotein E genotypes among diverse middle‐aged and older Latinos: study of Latinos‐investigation of neurocognitive aging results (HCHS/SOL). Sci Rep. 2018;8:17578. 30546063 10.1038/s41598-018-35573-3 PMC6292877

[42] Alexander DH , Novembre J , Lange K . Fast model‐based estimation of ancestry in unrelated individuals. Genome Res. 2009;19(9):1655‐1664. 19648217 10.1101/gr.094052.109 PMC2752134

[43] Cavalli‐Sforza LL . The human genome diversity project: past, present and future. Nat Rev Genet. 2005;6(4):333‐340. 15803201 10.1038/nrg1596

[44] McVean GA , Altshuler (Co‐Chair) DM , Durbin (Co‐Chair) RM , et al. An integrated map of genetic variation from 1,092 human genomes. Nature. 2012;491(7422):56‐65. 23128226 10.1038/nature11632 PMC3498066

[45] Browning SR , Grinde K , Plantinga A , et al. Local ancestry inference in a large US‐based Hispanic/Latino Study: Hispanic Community Health Study/Study of Latinos (HCHS/SOL). G3 (Bethesda). 2016;6(6):1525‐1534. 27172203 10.1534/g3.116.028779 PMC4889649

[46] Browning SR , Browning BL . Rapid and accurate haplotype phasing and missing‐data inference for whole‐genome association studies by use of localized haplotype clustering. Am J Hum Genet. 2007;81(5):1084‐1097. 17924348 10.1086/521987 PMC2265661

[47] Prins J, Process and Product Comparisons . In: NIST/SEMATECH e‐Handbook of Statistical Methods. NIST/SEMATECH. 2012 [cited 2025 May 29]. Available from: https://www.itl.nist.gov/div898/handbook/prc/section1/prc16.htm

[48] Caselli RJ , Dueck AC , Osborne D , et al. Longitudinal modeling of age‐related memory decline and the APOE ε4 effect. N Engl J Med. 2009;361(3):255‐263. 19605830 10.1056/NEJMoa0809437 PMC2928998

[49] Damotte V , van der Lee SJ , Chouraki V , et al. Plasma amyloid β levels are driven by genetic variants near APOE, BACE1, APP, PSEN2: a genome‐wide association study in over 12,000 non‐demented participants. Alzheimers Dement. 2021;17(10):1663‐1674. 34002480 10.1002/alz.12333 PMC8597077

[50] Cooper JG , Ghodsi M , Stukas S , Leach S , Brooks‐Wilson A , Wellington CL . APOE ε4 carrier status modifies plasma p‐tau181 concentrations in cognitively healthy super‐seniors. Alzheimers Dement. 2024;20(6):4373‐4380. 38752508 10.1002/alz.13804 PMC11180846

[51] Malek‐Ahmadi M , Su Y , Ghisays V , et al. Plasma NfL is associated with the APOE ε4 allele, brain imaging measurements of neurodegeneration, and lower recall memory scores in cognitively unimpaired late‐middle‐aged and older adults. Alzheimers Res Ther. 2023;15:74. 37038190 10.1186/s13195-023-01221-w PMC10084600

[52] Zhang C , Li B , Ng KP , et al. Plasma neurofilament light mediates the effects of apolipoprotein E on brain atrophy and cognitive decline in the comorbid Alzheimer's disease and cerebral small vessel disease. J Prev Alzheimers Dis. 2025;12(3):100054.

[53] Imbimbo BP , Watling M , Imbimbo C , Nisticò R . Plasma ATN(I) classification and precision pharmacology in Alzheimer's disease. Alzheimer's Dement. 2023;19:4729‐4734. 37079778 10.1002/alz.13084

[54] Simonsen AH , Gleerup HS , Musaeus CS , et al. Neurofilament light chain levels in serum among a large mixed memory clinic cohort: confounders and diagnostic usefulness. Alzheimers Dement Diagn Assess Dis Monit. 2023;15(4):e12512. 10.1002/dad2.12512 PMC10716567 38094990

[55] Syrjanen JA , Campbell MR , Algeciras‐Schimnich A , et al. Associations of amyloid and neurodegeneration plasma biomarkers with comorbidities. Alzheimers Dement J Alzheimers Assoc. 2022;18(6):1128‐1140. 10.1002/alz.12466 PMC8957642 34569696

[56] Griswold AJ , Celis K , Bussies P , et al. Increased APOEε4 expression is associated with the difference in Alzheimer disease risk from diverse ancestral backgrounds. Alzheimers Dement J Alzheimers Assoc. 2021;17(7):1179‐1188. 10.1002/alz.12287 PMC8843031 33522086

[57] Naslavsky MS , Suemoto CK , Brito LA , et al. Global and local ancestry modulate APOE association with Alzheimer's neuropathology and cognitive outcomes in an admixed sample. Mol Psychiatry. 2022;27(11):4800‐4808. 36071110 10.1038/s41380-022-01729-x PMC9734036

[58] Evans DA , Bennett DA , Wilson RS , et al. Incidence of Alzheimer disease in a Biracial Urban Community: relation to apolipoprotein E allele status. Arch Neurol. 2003;60(2):185. 12580702 10.1001/archneur.60.2.185

[59] Rajabli F , Feliciano BE , Celis K , et al. Ancestral origin of ApoE ε4 Alzheimer disease risk in Puerto Rican and African American populations. PLoS Genet. 2018;14(12):e1007791. 30517106 10.1371/journal.pgen.1007791 PMC6281216

[60] Blue EE , Horimoto ARVR , Mukherjee S , Wijsman EM , Thornton TA . Local ancestry at APOE modifies Alzheimer's disease risk in Caribbean Hispanics. Alzheimers Dement J Alzheimers Assoc. 2019;15(12):1524‐1532. 10.1016/j.jalz.2019.07.016 PMC6925639 31606368

[61] Kulminski AM , Jain‐Washburn E , Loiko E , Loika Y , Feng F , Culminskaya I . Associations of the APOE ε2 and ε4 alleles and polygenic profiles comprising APOE‐TOMM40‐APOC1 variants with Alzheimer's disease biomarkers. Aging. 2022;14(24):9782‐9804. 36399096 10.18632/aging.204384 PMC9831745

[62] Chatterjee P , Pedrini S , Ashton NJ , et al. Diagnostic and prognostic plasma biomarkers for preclinical Alzheimer's disease. Alzheimers Dement. 2022;18(6):1141‐1154. 34494715 10.1002/alz.12447

[63] Huang YY , Yang YX , Wang HF , et al. Genome‐wide association study identifies APOE locus influencing plasma p‐tau181 levels. J Hum Genet. 2022;67(8):459‐463. 35250029 10.1038/s10038-022-01026-z

[64] Contreras JA , Ortega NE , Espejo K , Aslanyan V , Pa J . The effect of APOE4 on Alzheimer's plasma biomarkers among Mexican Americans in the HABS‐HD cohort. Alzheimers Res Ther. 2025;17:208. 41029418 10.1186/s13195-025-01845-0 PMC12482669

[65] Wang Y , Li F , Qin Q , et al. Influence of APOE ε4 on performance of CSF biomarkers in differentiating clinical Alzheimer's disease. J Prev Alzheimers Dis. 2025;12(4):100065. 39827005 10.1016/j.tjpad.2025.100065 PMC12184029

[66] Weigand AJ , Ortiz G , Walker KS , Galasko DR , Bondi MW , Thomas KR . APOE differentially moderates cerebrospinal fluid and plasma phosphorylated tau181 associations with multi‐domain cognition. Neurobiol Aging. 2023;125:1‐8. 36780762 10.1016/j.neurobiolaging.2022.10.016

[67] Saeed A , Lopez O , Cohen A , Reis SE . Cardiovascular disease and Alzheimer's disease: the heart–brain axis. J Am Heart Assoc. 2023;12(21):e030780. 37929715 10.1161/JAHA.123.030780 PMC10727398

[68] Palix C , Felisatti F , Gonneaud J , et al. Relationships between diabetes‐related vascular risk factors and neurodegeneration biomarkers in healthy aging and Alzheimer's disease. Neurobiol Aging. 2022;118:25‐33. 35843110 10.1016/j.neurobiolaging.2022.06.004

[69] Van Gils V , Rizzo M , Côté J , et al. The association of glucose metabolism measures and diabetes status with Alzheimer's disease biomarkers of amyloid and tau: a systematic review and meta‐analysis. Neurosci Biobehav Rev. 2024;159:105604. 38423195 10.1016/j.neubiorev.2024.105604

[70] Yu F , Pituch KA , Maxfield M , et al. The associations between type 2 diabetes and plasma biomarkers of Alzheimer's disease in the Health and Aging Brain Study: Health Disparities (HABS‐HD). PLOS ONE. 2024;19(4):e0295749. 38558059 10.1371/journal.pone.0295749 PMC10984470

[71] Montoliu‐Gaya L , Bian S , Dammer EB , et al. Proteomic analysis of Down syndrome cerebrospinal fluid compared to late‐onset and autosomal dominant Alzheimer´s disease. Nat Commun. 2025;16(1):6003. 40595720 10.1038/s41467-025-61054-z PMC12214755

[72] Johnson ECB , Bian S , Haque RU , et al. Cerebrospinal fluid proteomics define the natural history of autosomal dominant Alzheimer's disease. Nat Med. 2023;29(8):1979‐1988. 37550416 10.1038/s41591-023-02476-4 PMC10427428

