# Supplementary material for: APOE and plasma AD biomarkers: The role of genetic ancestry in Hispanics/Latinos
Source: Alzheimers Dement. 2026 Mar 12;22(3):e71213. doi: 10.1002/alz.71213 (PMC13093554; doi:10.1002/alz.71213)
Supplement: Supplementary file 1 — Supplementary table S1. Coefficients of variation (CV%) for plasma ATN(I) biomarkers. Supplementary figure 1. Heatmap of unadjusted pairwise Spearman correlation coefficients between plasma ATN(I) biomarkers. Supplementary figure 2. Distributions of plasma ATN(I) biomarkers and model residuals. Supplementary table S2. Model fit comparison across nested primary analysis regression models using AIC. Supplementary table S3. Associations between ATN(I) biomarkers and APOE alleles based on additive inheritance mode. Supplementary table S4. Covariate associations with plasma ATN(I) biomarkers in primary analysis. Supplementary figure 3. Distributions of plasma ATN(I) biomarkers by age. Supplementary figure 4. Distribution of plasma ATN(I) biomarkers by sex. Supplementary table S5. Associations between ATN(I) biomarkers and APOE allele (additive mode) by age and sex. Supplementary table S6. Associations between ATN(I) biomarkers and APOE allele (additive mode) by age and sex, adjusted for global ancestry. Supplementary table S7. Associations between ATN(I) biomarkers and APOE allele (additive mode) by genetic analysis group. Supplementary table S8. Associations between ATN(I) biomarkers and APOE allele (additive mode) by genetic analysis group, adjusted for global ancestry. Supplementary table S9. Interaction of global genetic ancestry proportion and APOE alleles (additive) on ATN(I) biomarkers. Supplementary figure 5. Interaction of global genetic ancestry proportion and APOE ε2 allele on ATN(I) biomarkers. Supplementary table S10. Associations between ATN(I) biomarkers and APOE allele by local genetic ancestry. Supplementary figure 6. Interaction of local genetic ancestry counts and APOE ε2 allele on ATN(I) biomarkers. Supplementary table S11. Stratified interaction of local ancestry proportion and APOE alleles on ATN(I) biomarkers. Supplementary figure 7. Associations between ATN(I) biomarkers and APOE allele by global genetic ancestry, stratified by age. Supplementary [file ALZ-22-e71213-s002.docx]

**Supplementary table S1. Coefficients of variation (CV%) for plasma ATN(I) biomarkers.**

| Biomarker | Mean  (unweighted; pg/ml) | SD  (unweighted; pg/ml) | CV%  (this study) | Inter-assay CV% (Quanterix internal) |
| --- | --- | --- | --- | --- |
| Aꞵ40 | 115.18 | 23.60 | 20.49 | 4.2 |
| Aꞵ42 | 8.04 | 1.91 | 23.76 | 3.7 |
| pTau-181 | 1.76 | 0.73 | 41.73 | 7.2 |
| NfL | 16.44 | 7.85 | 47.76 | 10.3 |
| GFAP | 146.35 | 59.09 | 40.38 | 4.1 |

NOTE: The within-study was calculated using all available plasma biomarker data from study participants (n = 6,118), using the ratio of the standard deviation (SD) to the mean × 100%. Reported inter-assay CVs were obtained from Quanterix internal validation studies for the same assays from all SOL-INCA participants with available blood samples (n = 6,226).

**Supplementary figure 1. Heatmap of unadjusted pairwise Spearman correlation coefficients between plasma ATN(I) biomarkers.**

**
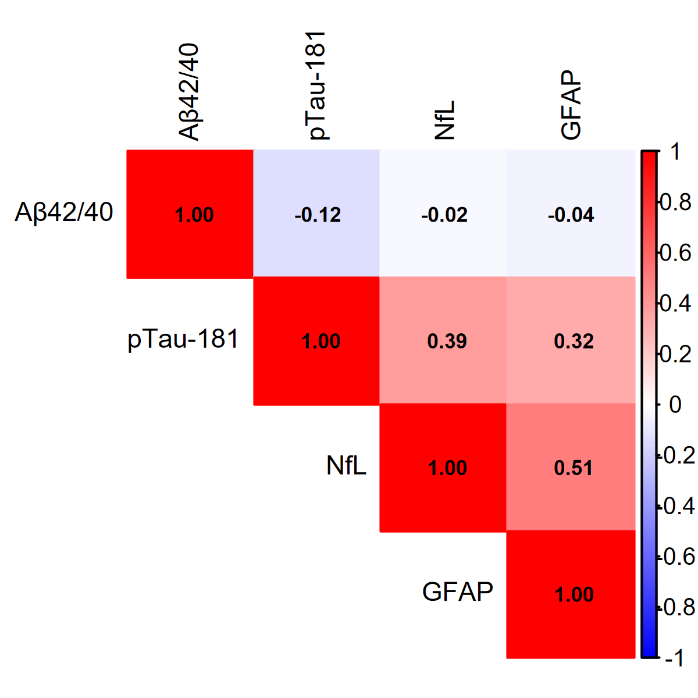
**

**Supplementary figure 2. Distributions of plasma ATN(I) biomarkers and model residuals.**


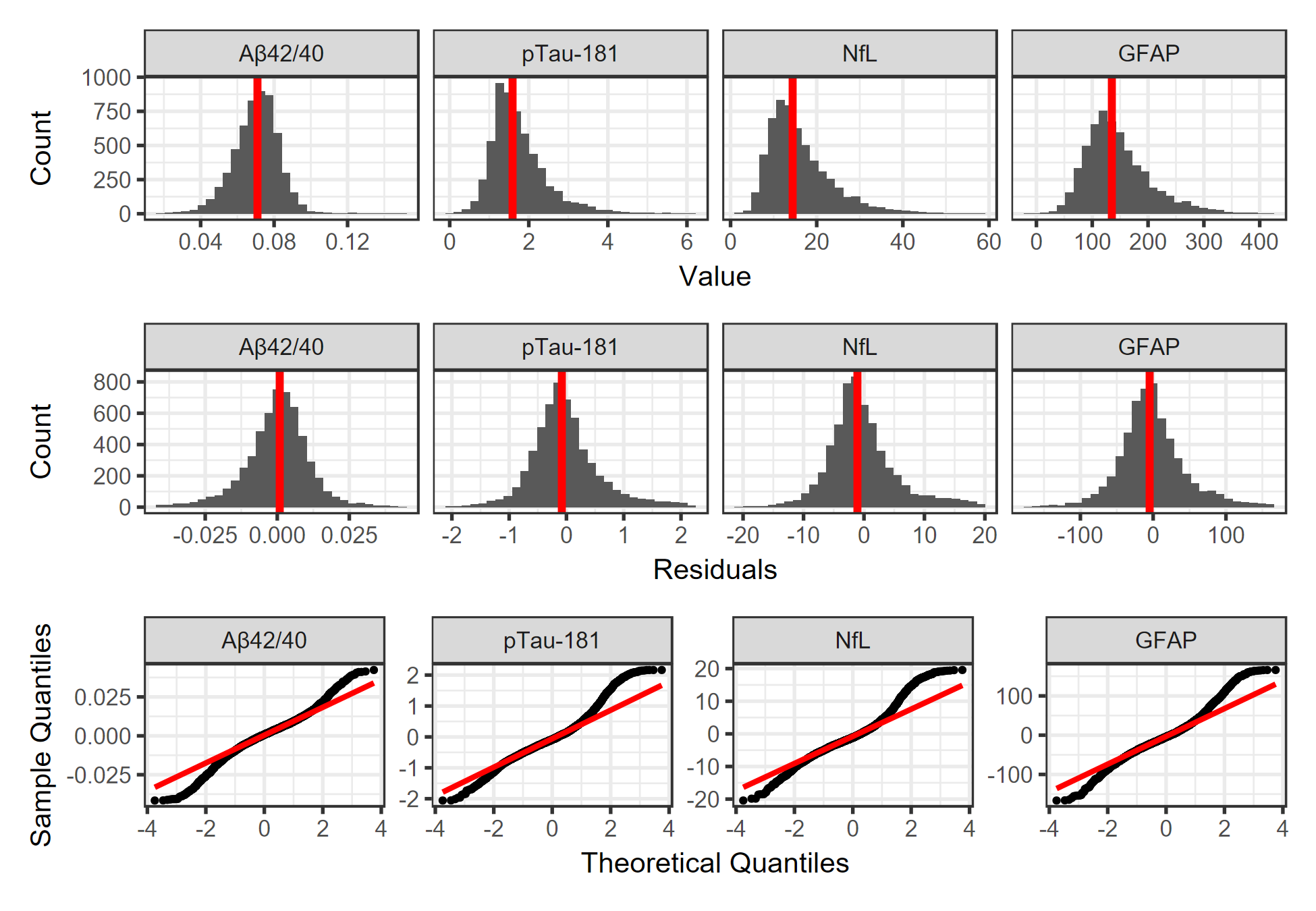


NOTE: Histograms show the distribution of raw biomarker values (top panel) and residuals from survey-weighted linear regression models including APOE ε2 and ε4 alleles, sex, age, and study center as covariates (middle panel). Red vertical lines indicate median values for each biomarker. QQ plots (bottom panel) display the residuals against theoretical quantiles to assess approximate normality. Red lines represent the expected values under a standard normal distribution. Residuals suggest that the regression model appropriately captures the central tendency of each biomarker, and the QQ plots indicate reasonable adherence to normality assumptions.

**Supplementary table S2. Model fit comparison across nested primary analysis regression models using AIC.**

| Model | **Model 0** | **Model 1** | **Model 2** |
| --- | --- | --- | --- |
| Covariates | Unadjusted | Age, sex, center | Age, sex, center, global genetic ancestry |
| Aꞵ42/40 | -26637 | -26915 | **-26926** |
| Ptau-181 | 9476 | 8739 | **8720** |
| Nfl | 30896 | **29126** | 29133 |
| GFAP | 48536 | 46406 | **46400** |

NOTE: Table values represent the Akaike Information Criterion (AIC) values for three nested survey-weighted regression models evaluating associations between plasma ATN(I) biomarkers and *APOE* alleles based on the additive inheritance mode, calculated in the 4,912 participants with complete data for age, sex, study center, and global genetic ancestry. Model 0 is unadjusted, Model 1 adjusts for age, sex, and study center, and Model 2 further includes global genetic ancestry proportions (African and Amerindian, with European as the reference). The lowest AIC value indicating better model fit is bolded for each ATN(I) biomarker outcome.

|  |  | Model 0 |  |
| --- | --- | --- | --- |
| ATN(I) biomarker | ***APOE* allele** | **Estimate [95% CI]** | ***P* value** |
| Aꞵ42/40 | ε2 | 0.038 [-0.073, 0.147] | .505 |
|  | ε4 | -0.207 [-0.276, -0.138] | <.001*** |
| pTau-181 | ε2 | 0.012 [-0.105, 0.130] | .836 |
|  | ε4 | 0.212 [0.123, 0.311] | <.001*** |
| NfL | ε2 | -0.083 [-0.192, 0.031] | .152 |
|  | ε4 | 0.010 [-0.068, 0.088] | .805 |
| GFAP | ε2 | 0.004 [-0.107, 0.114] | .946 |
|  | ε4 | 0.143 [0.058, 0.231] | .001** |

**Supplementary table S3. Associations between ATN(I) biomarkers and *APOE* alleles based on additive inheritance mode.**

|  |  | Model 1 | |
| --- | --- | --- | --- |
| ATN(I) biomarker | ***APOE* allele** | **Estimate [95% CI]** | ***P* value** |
| Aꞵ42/40 | ε2 | 0.040 [-0.065, 0.147] | .454 |
|  | ε4 | -0.233 [-0.293, -0.164] | <.001*** |
| pTau-181 | ε2 | 0.025 [-0.082, 0.133] | .648 |
|  | ε4 | 0.240 [0.155, 0.311] | <.001*** |
| NfL | ε2 | -0.066 [-0.153, 0.017] | .114 |
|  | ε4 | 0.029 [-0.029, 0.087] | .332 |
| GFAP | ε2 | -0.030 [-0.110, 0.051] | .465 |
|  | ε4 | 0.160 [0.100, 0.214] | <.001*** |
|  |  | **Model 2** | |
| ATN(I) biomarker | ***APOE* allele** | **Estimate [95% CI]** | ***P* value** |
| Aꞵ42/40 | ε2 | 0.009 [-0.103, 0.121] | .880 |
|  | ε4 | -0.233 [-0.302, -0.164] | <.001*** |
| pTau-181 | ε2 | 0.007 [-0.109, 0.124] | .901 |
|  | ε4 | 0.240 [0.141, 0.325] | <.001*** |
| NfL | ε2 | -0.059 [-0.153, 0.036] | .221 |
|  | ε4 | 0.010 [-0.060, 0.079] | .786 |
| GFAP | ε2 | -0.049 [-0.133, 0.035] | .249 |
|  | ε4 | 0.138 [0.072, 0.198] | <.001*** |

NOTE: Model 0 (n = 6,116) was unadjusted; Model 1 (n = 6,116) was adjusted for age, sex, and study center; Model 2 (n = 4,912) was adjusted for age, sex, study center, and global genetic ancestry proportions (African and Amerindian, with European as the reference given that global ancestry proportions sum to 1). *APOE* ε3 was used as the reference allele. Effect sizes were estimated using the complex survey design method, standardized to the survey-weighted SD of each biomarker in the overall cohort, and reported with 95% CIs. Asterisks indicate statistical significance: ****P* < .001, ***P* < .01, **P* < .05.

**Supplementary table S4. Covariate associations with plasma ATN(I) biomarkers in primary analysis.**

| Aꞵ42/40 | | |
| --- | --- | --- |
| Covariate | **Estimate [95% CI]** | ***P* value** |
| age | -0.00023 [-0.00029, -0.00018] | <.001*** |
| sexMale | -5.8e-06 [-0.00081, 0.00079] | .989 |
| centerChicago | 0.0016 [3.9e-05, 0.0031] | .045* |
| centerMiami | -0.0033 [-0.0045, -0.0021] | <.001*** |
| centerSanDiego | -0.0012 [-0.0026, 0.00018] | .089 |
| global African ancestry | 0.0051 [0.0019, 0.0083] | .002** |
| global Amerindian ancestry | 0.0011 [-0.0012, 0.0034] | .368 |
| pTau-181 | | |
| Covariate | **Estimate [95% CI]** | ***P* value** |
| age | 0.033 [0.030, 0.036] | <.001*** |
| sexMale | 0.10 [0.049, 0.15] | <.001*** |
| centerChicago | 0.0039 [-0.078, 0.085] | .925 |
| centerMiami | -0.0092 [-0.082, 0.064] | .804 |
| centerSanDiego | -0.0023 [-0.080, 0.075] | .954 |
| global African ancestry | -0.14 [-0.33, 0.055] | .162 |
| global Amerindian ancestry | -0.27 [-0.39, -0.16] | <.001*** |
| NfL | | |
| Covariate | **Estimate [95% CI]** | ***P* value** |
| age | 0.57 [0.53, 0.60] | <.001*** |
| sexMale | 0.24 [-0.27, 0.75] | .357 |
| centerChicago | 0.13 [-0.65, 0.91] | .748 |
| centerMiami | -1.5 [-2.2, -0.82] | <.001*** |
| centerSanDiego | 0.082 [-0.73, 0.89] | .843 |
| global African ancestry | -0.30 [-2.3, 1.7] | .768 |
| global Amerindian ancestry | -0.49 [-1.8, 0.79] | .452 |
| GFAP | | |
| Covariate | **Estimate [95% CI]** | ***P* value** |
| age | 4.4 [4.2, 4.6] | <.001*** |
| sexMale | -22 [-25, -18] | <.001*** |
| centerChicago | -15 [-21, -9.6] | <.001*** |
| centerMiami | -9.2 [-15, -3.9] | <.001*** |
| centerSanDiego | -1.9 [-7.9, 4.1] | .529 |
| global African ancestry | 6.3 [-8.4, 21] | .398 |
| global Amerindian ancestry | -12 [-23, -0.30] | .044* |

NOTE: Covariate associations are based on model 2 of the primary analysis. Model 2 (n = 4,912) is based on the additive inheritance mode and adjusts for age, sex, study center, and global ancestry proportions (African and Amerindian, with European as the reference given that global ancestry proportions sum to 1). Effect sizes were estimated using the complex survey design method, standardized to the survey-weighted SD of each biomarker in the overall cohort, and reported with 95% CIs. Asterisks indicate statistical significance: ****P* < .001, ***P* < .01, **P* < .05.

**Supplementary figure 3. Distributions of plasma ATN(I) biomarkers by age.**


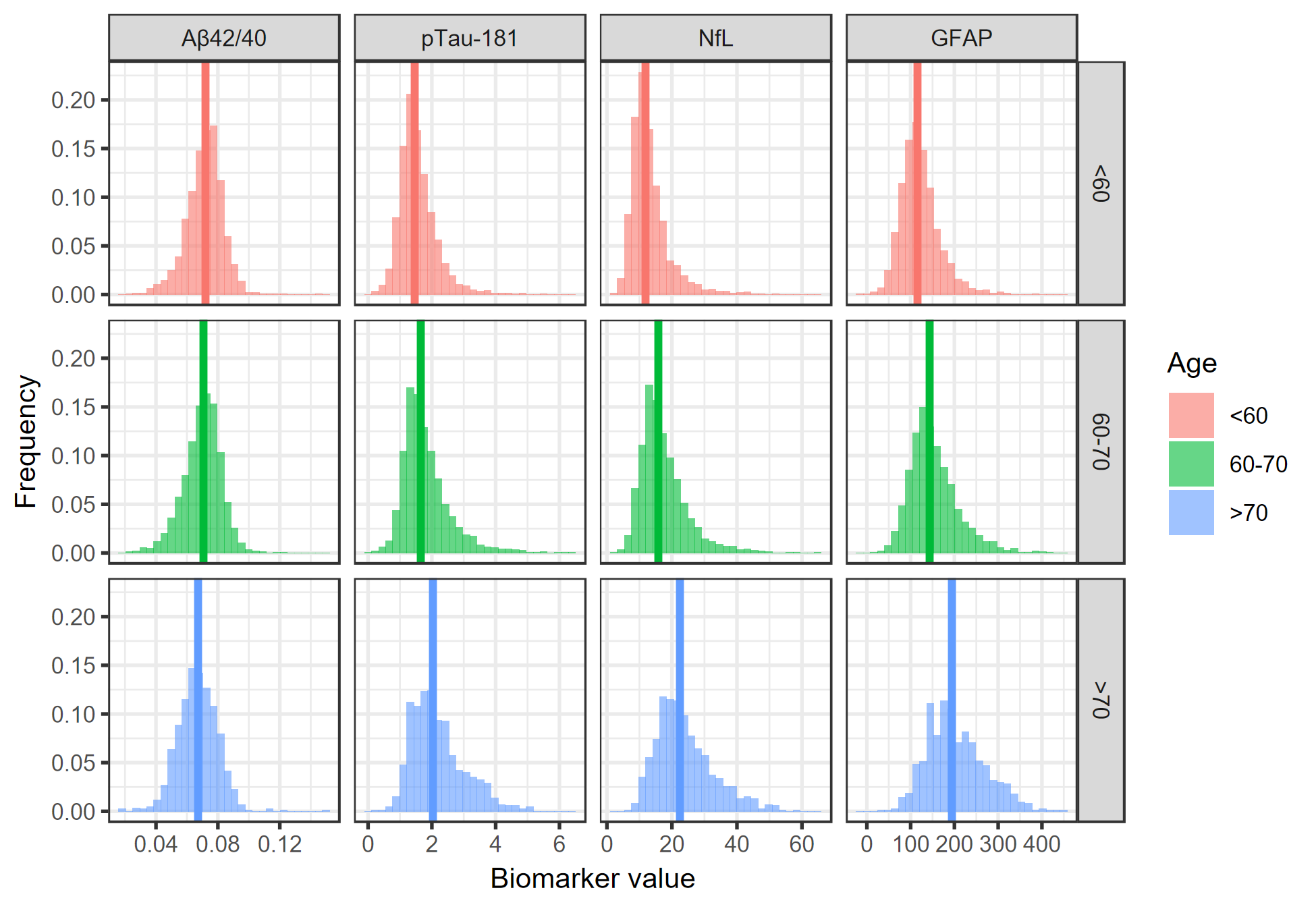


NOTE: Histograms show the distribution of raw plasma ATN biomarker values (Aβ42/40, pTau-181, NfL, GFAP) for each stratified age group (<60, 60-70, >70). Vertical lines indicate median biomarker values within the given age group.

**Supplementary figure 4. Distribution of plasma ATN(I) biomarkers by sex.**


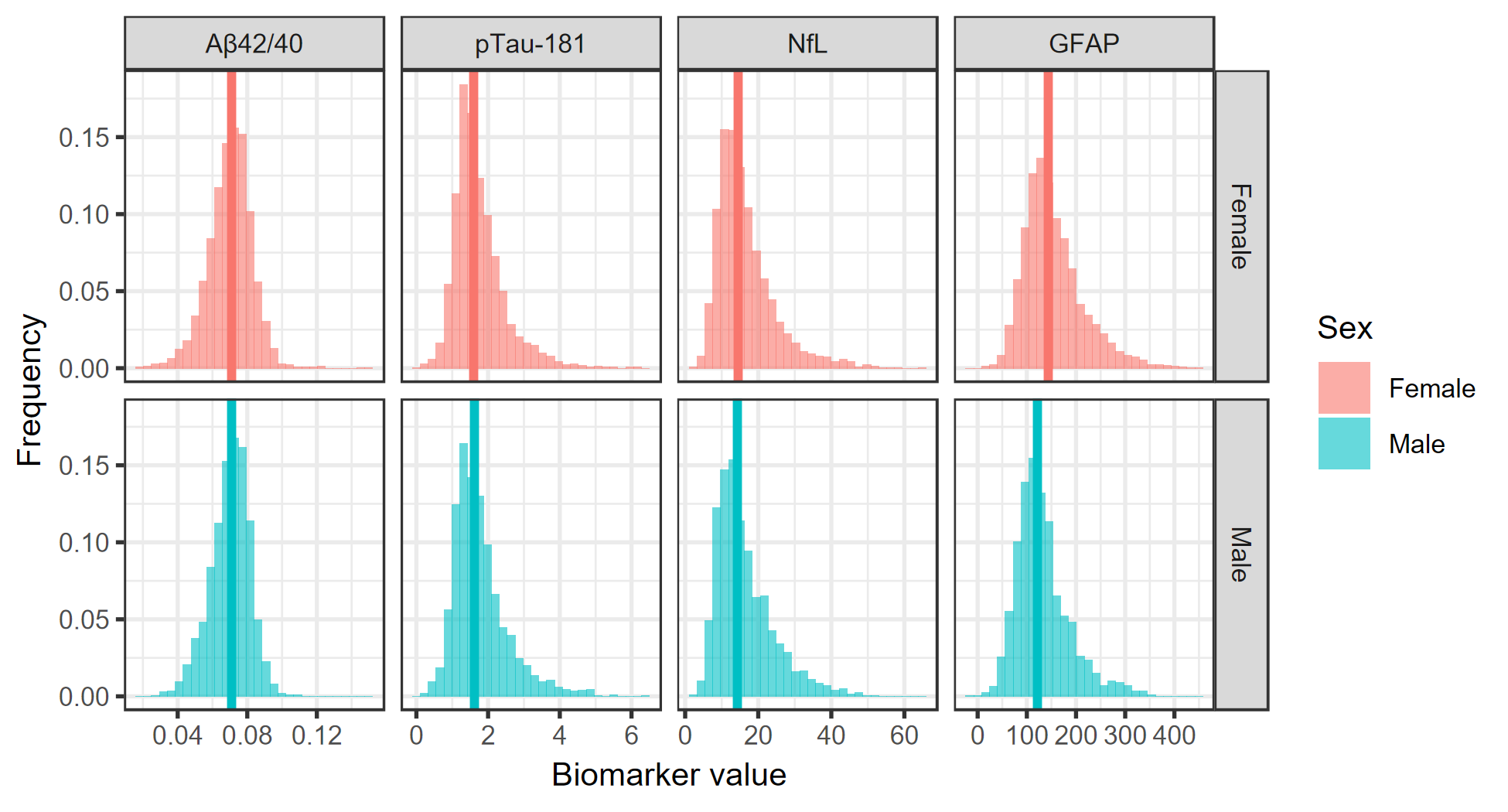


NOTE: Histograms show the distribution of raw plasma ATN biomarker values (Aβ42/40, pTau-181, NfL, GFAP) for each sex (Female, Male). Vertical lines indicate median biomarker values within the given sex.

**Supplementary table S5. Associations between ATN(I) biomarkers and *APOE* allele (additive mode) by age and sex.**

|  |  | | | <60 | |  | | |  | |  | |
| --- | --- | --- | --- | --- | --- | --- | --- | --- | --- | --- | --- | --- |
|  |  | | | **Female** | |  | **Male** | | | |  | |
| ATN(I) biomarker | ***APOE* allele** | | | **Estimate [95% CI]** | | ***P* value** | **Estimate [95% CI]** | | | | ***P* value** | |
| Aꞵ42/40 | ε2 | | | -0.036 [-0.207, 0.138] | | .370 | 0.086 [-0.112, 0.284] | | | | .268 | |
|  | ε4 | | | -0.129 [-0.241, -0.007] | | .062 | -0.198 [-0.379, -0.020] | | | | .026* | |
| pTau-181 | ε2 | | | 0.137 [-0.027, 0.297] | | .108 | -0.030 [-0.226, 0.169] | | | | .421 | |
|  | ε4 | | | 0.106 [-0.017, 0.226] | | .101 | 0.016 [-0.116, 0.141] | | | | .423 | |
| NfL | ε2 | | | 0.026 [-0.111, 0.166] | | .385 | -0.166 [-0.358, 0.024] | | | | .098 | |
|  | ε4 | | | 0.049 [-0.040, 0.140] | | .258 | -0.082 [-0.204, 0.041] | | | | .197 | |
| GFAP | ε2 | | | 0.063 [-0.071, 0.198] | | .268 | 0.058 [-0.117, 0.231] | | | | .318 | |
|  | ε4 | | | 0.028 [-0.082, 0.138] | | .351 | -0.043 [-0.155, 0.069] | | | | .325 | |
|  | |  | **60-70** | |  | | | | |  | |  |
|  | |  | **Female** | |  | | | **Male** | | | |  |
| ATN(I) biomarker | | ***APOE* allele** | **Estimate [95% CI]** | | ***P* value** | | | **Estimate [95% CI]** | | | | ***P* value** |
| Aꞵ42/40 | | ε2 | 0.121 [-0.095, 0.328] | | .166 | | | 0.233 [-0.036, 0.509] | | | | .059 |
|  | | ε4 | -0.267 [-0.397, -0.129] | | <.001*** | | | -0.164 [-0.328, -0.004] | | | | .080 |
| pTau-181 | | ε2 | 0.076 [-0.141, 0.297] | | .260 | | | -0.105 [-0.311, 0.103] | | | | .242 |
|  | | ε4 | 0.155 [0.021, 0.282] | | .020* | | | 0.138 [-0.052, 0.325] | | | | .106 |
| NfL | | ε2 | -0.065 [-0.230, 0.096] | | .294 | | | -0.166 [-0.358, 0.014] | | | | .118 |
|  | | ε4 | -0.033 [-0.128, 0.061] | | .325 | | | -0.038 [-0.166, 0.084] | | | | .371 |
| GFAP | | ε2 | -0.040 [-0.214, 0.133] | | .375 | | | -0.008 [-0.181, 0.165] | | | | .488 |
|  | | ε4 | 0.138 [0.030, 0.247] | | .029* | | | 0.231 [0.084, 0.379] | | | | .018* |
|  | |  | **>70** | |  | | | | |  | |  |
|  | |  | **Female** | |  | | | **Male** | | | |  |
| ATN(I) biomarker | | ***APOE* allele** | **Estimate [95% CI]** | | ***P* value** | | | **Estimate [95% CI]** | | | | ***P* value** |
| Aꞵ42/40 | | ε2 | -0.276 [-0.612, 0.055] | | .082 | | | -0.009 [-0.397, 0.379] | | | | .488 |
|  | | ε4 | -0.405 [-0.664, -0.155] | | .003** | | | -0.259 [-0.560, 0.034] | | | | .079 |
| pTau-181 | | ε2 | -0.020 [-0.381, 0.339] | | .479 | | | 0.155 [-0.297, 0.593] | | | | .259 |
|  | | ε4 | 0.579 [0.339, 0.819] | | <.001*** | | | 0.833 [0.494, 1.172] | | | | <.001*** |
| NfL | | ε2 | 0.102 [-0.217, 0.421] | | .279 | | | 0.089 [-0.396, 0.575] | | | | .347 |
|  | | ε4 | 0.255 [0.031, 0.485] | | .034* | | | 0.012 [-0.332, 0.345] | | | | .461 |
| GFAP | | ε2 | -0.036 [-0.379, 0.313] | | .435 | | | -0.247 [-0.643, 0.161] | | | | .166 |
|  | | ε4 | 0.527 [0.297, 0.758] | | <.001*** | | | 0.247 [0.003, 0.511] | | | | .074 |

NOTE: Models were adjusted for study center. Models are based on the additive inheritance mode with *APOE* ε3 used as the reference allele. Effect sizes were estimated using the complex survey design method, standardized to the survey-weighted SD of each biomarker in the overall cohort, and reported with 95% CIs. *P* values were estimated based on permutation testing with 10,000 permutations per age-sex group. Asterisks indicate statistical significance: ****P* < .001, ***P* < .01, **P* < .05.

**Supplementary table S6. Associations between ATN(I) biomarkers and *APOE* allele (additive mode) by age and sex, adjusted for global ancestry.**

|  |  | | | <60 | |  | | |  | |  | |
| --- | --- | --- | --- | --- | --- | --- | --- | --- | --- | --- | --- | --- |
|  |  | | | **Female** | |  | **Male** | | | |  | |
| ATN(I) biomarker | ***APOE* allele** | | | **Estimate [95% CI]** | | ***P* value** | **Estimate [95% CI]** | | | | ***P* value** | |
| Aꞵ42/40 | ε2 | | | -0.121 [-0.310, 0.062] | | .149 | 0.129 [-0.072, 0.336] | | | | .188 | |
|  | ε4 | | | -0.112 [-0.250, 0.020] | | .120 | -0.198 [-0.379, -0.021] | | | | .035* | |
| pTau-181 | ε2 | | | 0.083 [-0.092, 0.254] | | .234 | -0.035 [-0.240, 0.169] | | | | .426 | |
|  | ε4 | | | 0.061 [-0.064, 0.184] | | .242 | 0.051 [-0.095, 0.198] | | | | .315 | |
| NfL | ε2 | | | 0.036 [-0.097, 0.166] | | .355 | -0.192 [-0.396, 0.010] | | | | .072 | |
|  | ε4 | | | 0.023 [-0.075, 0.121] | | .385 | -0.166 [-0.307, -0.018] | | | | .047* | |
| GFAP | ε2 | | | 0.069 [-0.069, 0.214] | | .261 | 0.015 [-0.165, 0.198] | | | | .441 | |
|  | ε4 | | | 0.000 [-0.092, 0.094] | | .480 | -0.063 [-0.198, 0.064] | | | | .269 | |
|  | |  | **60-70** | |  | | | | |  | |  |
|  | |  | **Female** | |  | | | **Male** | | | |  |
| ATN(I) biomarker | | ***APOE* allele** | **Estimate [95% CI]** | | ***P* value** | | | **Estimate [95% CI]** | | | | ***P* value** |
| Aꞵ42/40 | | ε2 | 0.129 [-0.129, 0.388] | | .170 | | | 0.164 [-0.164, 0.491] | | | | .168 |
|  | | ε4 | -0.216 [-0.362, -0.071] | | .005** | | | -0.216 [-0.388, -0.034] | | | | .045* |
| pTau-181 | | ε2 | 0.049 [-0.226, 0.325] | | .348 | | | -0.085 [-0.325, 0.155] | | | | .317 |
|  | | ε4 | 0.066 [-0.068, 0.198] | | .207 | | | 0.120 [-0.085, 0.325] | | | | .151 |
| NfL | | ε2 | -0.068 [-0.243, 0.105] | | .303 | | | -0.106 [-0.332, 0.119] | | | | .257 |
|  | | ε4 | -0.007 [-0.117, 0.102] | | .479 | | | -0.041 [-0.179, 0.097] | | | | .370 |
| GFAP | | ε2 | -0.077 [-0.264, 0.107] | | .266 | | | 0.002 [-0.181, 0.181] | | | | .484 |
|  | | ε4 | 0.150 [0.033, 0.264] | | .031* | | | 0.247 [0.077, 0.412] | | | | .021* |
|  | |  | **>70** | |  | | | | |  | |  |
|  | |  | **Female** | |  | | | **Male** | | | |  |
| ATN(I) biomarker | | ***APOE* allele** | **Estimate [95% CI]** | | ***P* value** | | | **Estimate [95% CI]** | | | | ***P* value** |
| Aꞵ42/40 | | ε2 | -0.276 [-0.629, 0.072] | | .105 | | | 0.054 [-0.345, 0.448] | | | | .430 |
|  | | ε4 | -0.440 [-0.716, -0.164] | | .004** | | | -0.284 [-0.595, 0.030] | | | | .077 |
| pTau-181 | | ε2 | -0.138 [-0.480, 0.198] | | .266 | | | 0.155 [-0.282, 0.593] | | | | .269 |
|  | | ε4 | 0.664 [0.381, 0.932] | | <.001*** | | | 0.847 [0.494, 1.201] | | | | <.001*** |
| NfL | | ε2 | 0.105 [-0.243, 0.460] | | .286 | | | 0.037 [-0.511, 0.587] | | | | .420 |
|  | | ε4 | 0.255 [-0.012, 0.511] | | .051 | | | -0.002 [-0.383, 0.370] | | | | .516 |
| GFAP | | ε2 | -0.109 [-0.461, 0.247] | | .303 | | | -0.264 [-0.659, 0.145] | | | | .159 |
|  | | ε4 | 0.527 [0.264, 0.807] | | <.001*** | | | 0.163 [-0.105, 0.428] | | | | .172 |

NOTE: Models were adjusted for study center and global genetic ancestry proportions (African and Amerindian, with European as the reference). Models are based on the additive inheritance mode with *APOE* ε3 used as the reference allele. Effect sizes were estimated using the complex survey design method, standardized to the survey-weighted SD of each biomarker in the overall cohort, and reported with 95% CIs. *P* values were estimated based on permutation testing with 10,000 permutations per age-sex group. Asterisks indicate statistical significance: ****P* < .001, ***P* < .01, **P* < .05.

**Supplementary table S7. Associations between ATN(I) biomarkers and *APOE* allele (additive mode) by genetic analysis group.**

|  |  | | | **Central American (n = 482)** | | | **Cuban (n = 977)** | | | |
| --- | --- | --- | --- | --- | --- | --- | --- | --- | --- | --- |
| ATN(I) biomarker | ***APOE* allele** | | | **Estimate [95% CI]** | | ***P* value** | **Estimate [95% CI]** | | ***P* value** | |
| Aꞵ42/40 | ε2 | | | -0.526 [-0.948, -0.103] | | .016* | -0.086 [-0.276, 0.095] | | .235 | |
|  | ε4 | | | -0.121 [-0.371, 0.129] | | .197 | -0.276 [-0.397, -0.147] | | .003** | |
| pTau-181 | ε2 | | | 0.141 [-0.325, 0.621] | | .254 | -0.107 [-0.311, 0.100] | | .180 | |
|  | ε4 | | | 0.325 [0.068, 0.565] | | .015* | 0.325 [0.155, 0.508] | | <.001*** | |
| NfL | ε2 | | | 0.052 [-0.179, 0.281] | | .388 | -0.140 [-0.294, 0.022] | | .098 | |
|  | ε4 | | | 0.111 [-0.230, 0.460] | | .192 | 0.056 [-0.102, 0.217] | | .267 | |
| GFAP | ε2 | | | -0.329 [-0.610, -0.040] | | .054 | -0.036 [-0.181, 0.107] | | .373 | |
|  | ε4 | | | 0.102 [-0.150, 0.346] | | .216 | 0.231 [0.096, 0.379] | | .008** | |
|  | |  | **Dominican (n = 465)** | | | | | **Mexican (n = 1732)** | | |
| ATN(I) biomarker | | ***APOE* allele** | **Estimate [95% CI]** | | ***P* value** | | | **Estimate [95% CI]** | | ***P* value** |
| Aꞵ42/40 | | ε2 | 0.066 [-0.172, 0.302] | | .335 | | | 0.397 [0.181, 0.603] | | .002** |
|  | | ε4 | -0.216 [-0.414, -0.017] | | .035* | | | -0.190 [-0.310, -0.072] | | .027* |
| pTau-181 | | ε2 | 0.079 [-0.169, 0.325] | | .293 | | | 0.058 [-0.141, 0.254] | | .314 |
|  | | ε4 | -0.005 [-0.184, 0.184] | | .488 | | | 0.240 [0.095, 0.395] | | .012* |
| NfL | | ε2 | 0.066 [-0.179, 0.307] | | .317 | | | -0.041 [-0.243, 0.153] | | .396 |
|  | | ε4 | -0.040 [-0.204, 0.126] | | .364 | | | 0.014 [-0.080, 0.107] | | .431 |
| GFAP | | ε2 | 0.063 [-0.150, 0.280] | | .325 | | | -0.033 [-0.214, 0.142] | | .416 |
|  | | ε4 | -0.049 [-0.198, 0.107] | | .338 | | | 0.157 [0.066, 0.247] | | .045* |
|  | |  | **Puerto Rican (n = 856)** | | | | | **South American (n = 381)** | | |
| ATN(I) biomarker | | ***APOE* allele** | **Estimate [95% CI]** | | ***P* value** | | | **Estimate [95% CI]** | | ***P* value** |
| Aꞵ42/40 | | ε2 | 0.049 [-0.267, 0.362] | | .393 | | | -0.241 [-0.853, 0.362] | | .178 |
|  | | ε4 | -0.302 [-0.509, -0.103] | | .002** | | | -0.233 [-0.526, 0.070] | | .089 |
| pTau-181 | | ε2 | 0.134 [-0.083, 0.353] | | .198 | | | -0.120 [-0.438, 0.198] | | .345 |
|  | | ε4 | 0.339 [0.109, 0.565] | | .001** | | | -0.023 [-0.254, 0.212] | | .470 |
| NfL | | ε2 | -0.033 [-0.217, 0.153] | | .440 | | | -0.319 [-0.664, 0.011] | | .094 |
|  | | ε4 | -0.029 [-0.192, 0.125] | | .380 | | | -0.098 [-0.319, 0.119] | | .273 |
| GFAP | | ε2 | -0.076 [-0.280, 0.125] | | .318 | | | -0.280 [-0.560, -0.006] | | .125 |
|  | | ε4 | 0.198 [0.021, 0.379] | | .024* | | | -0.100 [-0.264, 0.061] | | .261 |

NOTE: Models were adjusted for age, sex, and study center. Models are based on the additive inheritance mode with *APOE* ε3 used as the reference allele. Effect sizes were estimated using the complex survey design method, standardized to the survey-weighted SD of each biomarker in the overall cohort, and reported with 95% CIs. *P* values were estimated based on permutation testing with 10,000 permutations per genetic analysis group. Asterisks indicate statistical significance: ****P* < .001, ***P* < .01, **P* < .05.

**Supplementary table S8. Associations between ATN(I) biomarkers and *APOE* allele (additive mode) by genetic analysis group, adjusted for global ancestry.**

|  |  | | | **Central American (n = 482)** | | | | **Cuban (n = 977)** | | | |
| --- | --- | --- | --- | --- | --- | --- | --- | --- | --- | --- | --- |
| ATN(I) biomarker | ***APOE* allele** | | | **Estimate [95% CI]** | | | ***P* value** | **Estimate [95% CI]** | | ***P* value** | |
| Aꞵ42/40 | ε2 | | | -0.543 [-0.948, -0.121] | | | .012* | -0.095 [-0.284, 0.086] | | .207 | |
|  | ε4 | | | -0.129 [-0.371, 0.121] | | | .184 | -0.284 [-0.405, -0.155] | | .003** | |
| pTau-181 | ε2 | | | 0.123 [-0.353, 0.593] | | | .279 | -0.100 [-0.311, 0.112] | | .193 | |
|  | ε4 | | | 0.311 [0.071, 0.565] | | | .016* | 0.339 [0.155, 0.523] | | .001** | |
| NfL | ε2 | | | 0.057 [-0.179, 0.294] | | | .375 | -0.140 [-0.294, 0.017] | | .097 | |
|  | ε4 | | | 0.114 [-0.230, 0.447] | | | .185 | 0.054 [-0.107, 0.217] | | .278 | |
| GFAP | ε2 | | | -0.313 [-0.593, -0.012] | | | .067 | -0.046 [-0.181, 0.096] | | .344 | |
|  | ε4 | | | 0.104 [-0.147, 0.346] | | | .210 | 0.231 [0.086, 0.362] | | .009** | |
|  | |  | **Dominican (n = 465)** | | | | | | **Mexican (n = 1732)** | | |
| ATN(I) biomarker | | ***APOE* allele** | **Estimate [95% CI]** | | | ***P* value** | | | **Estimate [95% CI]** | | ***P* value** |
| Aꞵ42/40 | | ε2 | 0.049 [-0.190, 0.293] | | | .390 | | | 0.388 [0.181, 0.603] | | .002** |
|  | | ε4 | -0.216 [-0.414, -0.019] | | | .037* | | | -0.190 [-0.310, -0.074] | | .026* |
| pTau-181 | | ε2 | 0.079 [-0.184, 0.339] | | | .306 | | | 0.052 [-0.155, 0.254] | | .333 |
|  | | ε4 | 0.003 [-0.184, 0.184] | | | .487 | | | 0.240 [0.095, 0.395] | | .008** |
| NfL | | ε2 | 0.083 [-0.166, 0.332] | | | .286 | | | -0.040 [-0.243, 0.153] | | .397 |
|  | | ε4 | -0.038 [-0.204, 0.128] | | | .368 | | | 0.014 [-0.080, 0.109] | | .426 |
| GFAP | | ε2 | 0.079 [-0.135, 0.297] | | | .284 | | | -0.038 [-0.214, 0.137] | | .390 |
|  | | ε4 | -0.048 [-0.198, 0.107] | | | .336 | | | 0.157 [0.066, 0.247] | | .046* |
|  | |  | | | **Puerto Rican (n = 856)** | | | | **South American (n = 381)** | | |
| ATN(I) biomarker | | ***APOE* allele** | | | **Estimate [95% CI]** | ***P* value** | | | **Estimate [95% CI]** | | ***P* value** |
| Aꞵ42/40 | | ε2 | | | 0.025 [-0.293, 0.345] | .454 | | | -0.241 [-0.862, 0.397] | | .189 |
|  | | ε4 | | | -0.319 [-0.517, -0.112] | .002** | | | -0.224 [-0.526, 0.080] | | .091 |
| pTau-181 | | ε2 | | | 0.169 [-0.058, 0.381] | .152 | | | -0.169 [-0.466, 0.134] | | .264 |
|  | | ε4 | | | 0.339 [0.114, 0.565] | <.001*** | | | -0.017 [-0.240, 0.212] | | .471 |
| NfL | | ε2 | | | -0.041 [-0.230, 0.153] | .415 | | | -0.396 [-0.741, -0.045] | | .052 |
|  | | ε4 | | | -0.031 [-0.192, 0.126] | .374 | | | -0.078 [-0.281, 0.126] | | .316 |
| GFAP | | ε2 | | | -0.049 [-0.264, 0.163] | .385 | | | -0.297 [-0.577, -0.007] | | .119 |
|  | | ε4 | | | 0.198 [0.025, 0.379] | .024* | | | -0.092 [-0.264, 0.071] | | .290 |

NOTE: Models were adjusted for age, sex, study center, and global genetic ancestry proportions (African and Amerindian, with European as the reference). Models are based on the additive inheritance mode with *APOE* ε3 used as the reference allele. Effect sizes were estimated using the complex survey design method, standardized to the survey-weighted SD of each biomarker in the overall cohort, and reported with 95% CIs. *P* values were estimated based on permutation testing with 10,000 permutations per genetic background group. Asterisks indicate statistical significance: ****P* < .001, ***P* < .01, **P* < .05.

**Supplementary table S9. Interaction of global genetic ancestry proportion and *APOE* alleles (additive) on ATN(I) biomarkers.**

|  |  | Aβ42/40 |  | |  |  |
| --- | --- | --- | --- | --- | --- | --- |
|  |  | ***APOE* ε2** |  | ***APOE* ε4** | |  |
| **Ancestry** | **Proportion** | **Estimate [95% CI]** | ***P* value** | **Estimate [95% CI]** | | ***P* value** |
| **African** | 0 | -0.039 [-0.207, 0.128] | .641 | -0.240 [-0.335, -0.144] | | <.001*** |
|  | 0.2 | -0.002 [-0.121, 0.116] | .970 | -0.233 [-0.307, -0.159] | | <.001*** |
|  | 0.4 | 0.035 [-0.107, 0.176] | .624 | -0.227 [-0.341, -0.112] | | <.001*** |
|  | 0.6 | 0.071 [-0.142, 0.284] | .503 | -0.221 [-0.400, -0.041] | | .014* |
|  | 0.8 | 0.109 [-0.193, 0.409] | .473 | -0.215 [-0.465, 0.036] | | .087 |
|  | 1 | 0.145 [-0.250, 0.541] | .463 | -0.208 [-0.531, 0.116] | | .198 |
| **Amerindian** | 0 | 0.048 [-0.122, 0.218] | .569 | -0.227 [-0.334, -0.118] | | <.001*** |
|  | 0.2 | 0.032 [-0.087, 0.152] | .590 | -0.223 [-0.297, -0.149] | | <.001*** |
|  | 0.4 | 0.016 [-0.191, 0.223] | .877 | -0.220 [-0.304, -0.135] | | <.001*** |
|  | 0.6 | -0.000 [-0.339, 0.339] | .999 | -0.216 [-0.345, -0.088] | | <.001*** |
|  | 0.8 | -0.016 [-0.496, 0.464] | .946 | -0.213 [-0.397, -0.029] | | .020* |
|  | 1 | -0.032 [-0.656, 0.591] | .917 | -0.209 [-0.453, 0.033] | | .084 |
| **European** | 0 | 0.212 [-0.116, 0.540] | .196 | -0.141 [-0.345, 0.064] | | .170 |
|  | 0.2 | 0.153 [-0.080, 0.385] | .188 | -0.168 [-0.313, -0.024] | | .020* |
|  | 0.4 | 0.094 [-0.057, 0.245] | .213 | -0.197 [-0.290, -0.103] | | <.001*** |
|  | 0.6 | 0.035 [-0.082, 0.152] | .550 | -0.225 [-0.297, -0.152] | | <.001*** |
|  | 0.8 | -0.024 [-0.188, 0.140] | .768 | -0.253 [-0.357, -0.149] | | <.001*** |
|  | 1 | -0.083 [-0.333, 0.166] | .505 | -0.281 [-0.440, -0.122] | | <.001*** |
|  |  | **pTau-181** |  | |  |  |
|  |  | ***APOE* ε2** |  | ***APOE* ε4** | |  |
| **Ancestry** | **Proportion** | **Estimate [95% CI]** | ***P* value** | **Estimate [95% CI]** | | ***P* value** |
| **African** | 0 | 0.084 [-0.071, 0.239] | .279 | 0.319 [0.201, 0.438] | | <.001*** |
|  | 0.2 | 0.025 [-0.096, 0.147] | .682 | 0.220 [0.133, 0.308] | | <.001*** |
|  | 0.4 | -0.034 [-0.165, 0.096] | .600 | 0.122 [0.007, 0.236] | | .034* |
|  | 0.6 | -0.093 [-0.270, 0.083] | .289 | 0.023 [-0.153, 0.199] | | .793 |
|  | 0.8 | -0.153 [-0.390, 0.085] | .200 | -0.076 [-0.322, 0.171] | | .540 |
|  | 1 | -0.212 [-0.518, 0.095] | .167 | -0.174 [-0.494, 0.145] | | .277 |
| **Amerindian** | 0 | -0.035 [-0.194, 0.124] | .659 | 0.236 [0.102, 0.369] | | <.001*** |
|  | 0.2 | 0.006 [-0.107, 0.120] | .912 | 0.232 [0.138, 0.325] | | <.001*** |
|  | 0.4 | 0.048 [-0.107, 0.202] | .537 | 0.227 [0.131, 0.322] | | <.001*** |
|  | 0.6 | 0.089 [-0.153, 0.331] | .460 | 0.223 [0.084, 0.362] | | <.001** |
|  | 0.8 | 0.130 [-0.210, 0.470] | .443 | 0.219 [0.021, 0.417] | | .027* |
|  | 1 | 0.172 [-0.271, 0.616] | .439 | 0.215 [-0.047, 0.476] | | .101 |
| **European** | 0 | 0.016 [-0.251, 0.282] | .906 | 0.024 [-0.202, 0.250] | | .832 |
|  | 0.2 | 0.018 [-0.171, 0.208] | .846 | 0.099 [-0.056, 0.256] | | .202 |
|  | 0.4 | 0.021 [-0.107, 0.148] | .742 | 0.175 [0.075, 0.274] | | <.001*** |
|  | 0.6 | 0.024 [-0.091, 0.138] | .679 | 0.250 [0.161, 0.339] | | <.001*** |
|  | 0.8 | 0.026 [-0.136, 0.188] | .745 | 0.325 [0.191, 0.460] | | <.001*** |
|  | 1 | 0.029 [-0.206, 0.264] | .806 | 0.401 [0.199, 0.603] | | <.001*** |
|  |  | **NfL** |  | |  |  |
|  |  | ***APOE* ε2** |  | ***APOE* ε4** | |  |
| **Ancestry** | **Proportion** | **Estimate [95% CI]** | ***P* value** | **Estimate [95% CI]** | | ***P* value** |
| **African** | 0 | -0.091 [-0.218, 0.036] | .151 | 0.055 [-0.038, 0.148] | | .237 |
|  | 0.2 | -0.065 [-0.156, 0.026] | .152 | 0.005 [-0.067, 0.077] | | .890 |
|  | 0.4 | -0.039 [-0.184, 0.105] | .587 | -0.045 [-0.156, 0.066] | | .416 |
|  | 0.6 | -0.013 [-0.245, 0.220] | .909 | -0.095 [-0.267, 0.077] | | .270 |
|  | 0.8 | 0.013 [-0.315, 0.341] | .938 | -0.144 [-0.384, 0.095] | | .226 |
|  | 1 | 0.039 [-0.388, 0.465] | .856 | -0.195 [-0.503, 0.114] | | .207 |
| **Amerindian** | 0 | -0.050 [-0.180, 0.081] | .449 | 0.014 [-0.101, 0.129] | | .811 |
|  | 0.2 | -0.064 [-0.158, 0.031] | .176 | 0.010 [-0.066, 0.085] | | .799 |
|  | 0.4 | -0.079 [-0.223, 0.066] | .276 | 0.005 [-0.065, 0.076] | | .880 |
|  | 0.6 | -0.093 [-0.323, 0.137] | .417 | 0.001 [-0.105, 0.107] | | .983 |
|  | 0.8 | -0.108 [-0.432, 0.216] | .506 | -0.003 [-0.160, 0.153] | | .968 |
|  | 1 | -0.122 [-0.543, 0.299] | .561 | -0.007 [-0.218, 0.204] | | .945 |
| **European** | 0 | 0.022 [-0.287, 0.331] | .886 | -0.120 [-0.307, 0.067] | | .200 |
|  | 0.2 | -0.004 [-0.229, 0.221] | .973 | -0.075 [-0.202, 0.052] | | .237 |
|  | 0.4 | -0.030 [-0.178, 0.118] | .688 | -0.030 [-0.109, 0.049] | | .445 |
|  | 0.6 | -0.055 [-0.151, 0.039] | .241 | 0.015 [-0.057, 0.087] | | .677 |
|  | 0.8 | -0.081 [-0.192, 0.029] | .140 | 0.060 [-0.054, 0.174] | | .294 |
|  | 1 | -0.107 [-0.284, 0.070] | .226 | 0.105 [-0.068, 0.278] | | .225 |
|  |  | **GFAP** |  | |  |  |
|  |  | ***APOE* ε2** |  | ***APOE* ε4** | |  |
| **Ancestry** | **Proportion** | **Estimate [95% CI]** | ***P* value** | **Estimate [95% CI]** | | ***P* value** |
| **African** | 0 | -0.060 [-0.180, 0.059] | .311 | 0.166 [0.084, 0.247] | | <.001*** |
|  | 0.2 | -0.047 [-0.136, 0.042] | .290 | 0.138 [0.067, 0.209] | | <.001*** |
|  | 0.4 | -0.034 [-0.134, 0.067] | .503 | 0.109 [-0.028, 0.247] | | .110 |
|  | 0.6 | -0.020 [-0.164, 0.123] | .779 | 0.081 [-0.138, 0.300] | | .458 |
|  | 0.8 | -0.007 [-0.206, 0.193] | .946 | 0.053 [-0.250, 0.357] | | .727 |
|  | 1 | 0.007 [-0.252, 0.265] | .960 | 0.025 [-0.366, 0.415] | | .899 |
| **Amerindian** | 0 | 0.007 [-0.118, 0.131] | .916 | 0.154 [0.040, 0.269] | | .007** |
|  | 0.2 | -0.058 [-0.141, 0.025] | .164 | 0.143 [0.071, 0.216] | | <.001*** |
|  | 0.4 | -0.122 [-0.249, 0.006] | .056 | 0.132 [0.064, 0.201] | | <.001*** |
|  | 0.6 | -0.186 [-0.395, 0.023] | .075 | 0.122 [0.015, 0.229] | | .023* |
|  | 0.8 | -0.250 [-0.549, 0.048] | .094 | 0.111 [-0.048, 0.270] | | .163 |
|  | 1 | -0.315 [-0.705, 0.076] | .107 | 0.100 [-0.115, 0.316] | | .353 |
| **European** | 0 | 0.010 [-0.199, 0.219] | .925 | 0.089 [-0.123, 0.300] | | .401 |
|  | 0.2 | -0.003 [-0.152, 0.147] | .972 | 0.109 [-0.042, 0.260] | | .147 |
|  | 0.4 | -0.015 [-0.116, 0.086] | .765 | 0.130 [0.033, 0.227] | | .007** |
|  | 0.6 | -0.028 [-0.114, 0.059] | .524 | 0.150 [0.084, 0.217] | | <.001*** |
|  | 0.8 | -0.040 [-0.158, 0.078] | .499 | 0.171 [0.082, 0.260] | | <.001*** |
|  | 1 | -0.053 [-0.226, 0.120] | .544 | 0.191 [0.050, 0.333] | | .007** |

NOTE: Estimates (in SD units) are derived from linear combinations of regression coefficients, adjusting for age, sex, and center. Asterisks indicate statistical significance: ****P* < .001, ***P* < .01, **P* < .05.

**Supplementary figure 5. Interaction of global genetic ancestry proportion and *APOE* ε2 allele on ATN(I) biomarkers.**

**
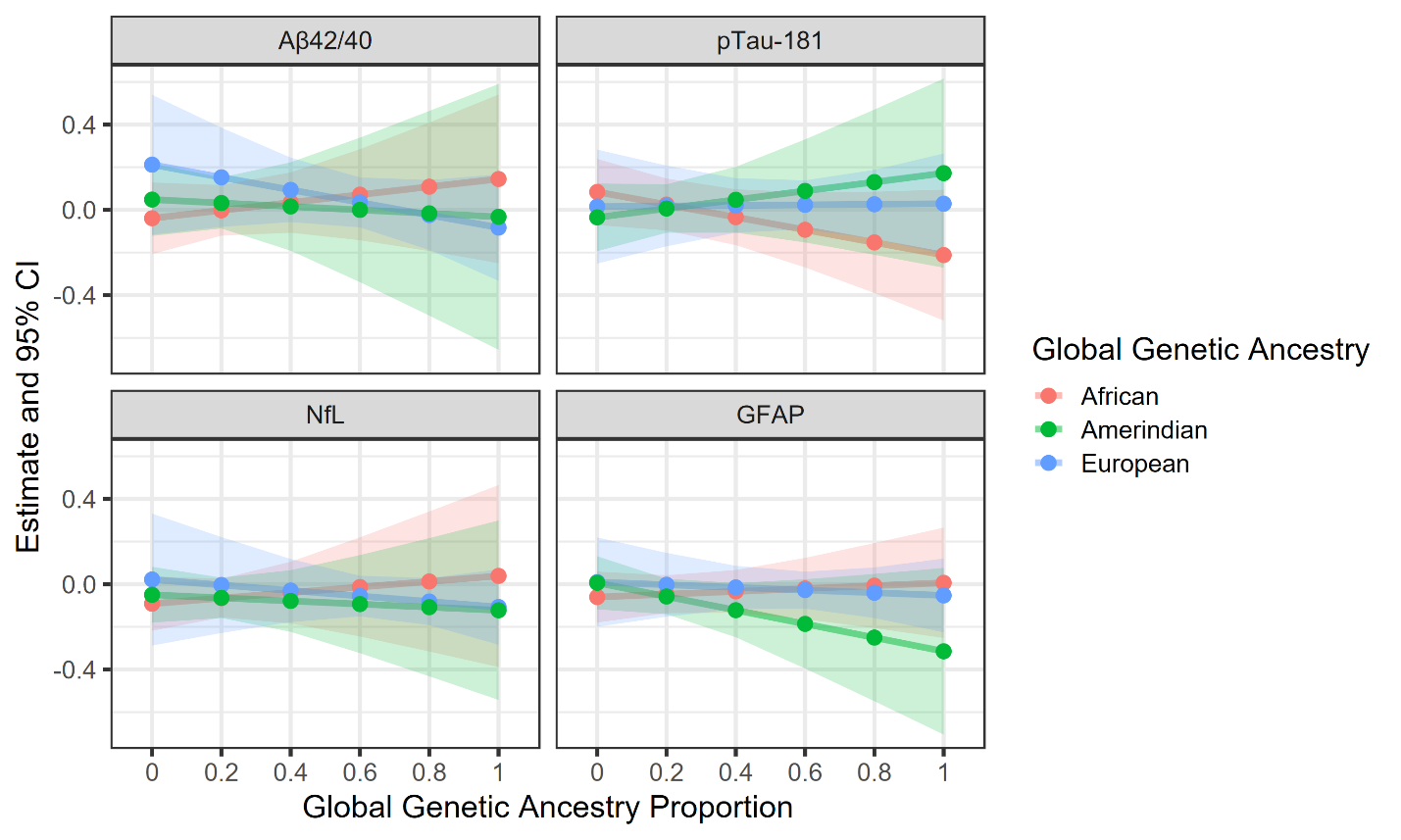
**

NOTE: Associations were tested at ancestry proportions of 0, 0.2, 0.4, 0.8, and 1, with effect estimates represented by points and shaded areas indicating 95% confidence intervals. Estimates were derived from linear combinations of regression coefficients, adjusting for age, sex, and study center, and were standardized to the survey-weighted SD of each biomarker in the overall cohort.

**Supplementary table S10. Associations between ATN(I) biomarkers and *APOE* allele by local genetic ancestry.**

|  |  | African |  | | | | |  |  |
| --- | --- | --- | --- | --- | --- | --- | --- | --- | --- |
|  |  | ***APOE* × Ancestry Interaction** | | | ***APOE* Main Effect** | | | |  |
| ATN(I) biomarker | ***APOE* allele** | **Estimate [95% CI]** | ***P* value** | | **Estimate [95% CI]** | | | | ***P* value** |
| Aꞵ42/40 | ε2 | 0.076 [-0.224, 0.371] | .341 | | -0.016 [-0.172, 0.147] | | | | .421 |
|  | ε4 | 0.103 [-0.112, 0.310] | .238 | | -0.259 [-0.345, -0.172] | | | | <.001*** |
| pTau-181 | ε2 | -0.226 [-0.508, 0.059] | .107 | | 0.076 [-0.079, 0.226] | | | | .182 |
|  | ε4 | -0.116 [-0.367, 0.133] | .215 | | 0.268 [0.155, 0.367] | | | | <.001*** |
| NfL | ε2 | 0.140 [-0.126, 0.409] | .204 | | -0.111 [-0.217, -0.003] | | | | .080 |
|  | ε4 | -0.095 [-0.294, 0.106] | .252 | | 0.029 [-0.060, 0.117] | | | | .305 |
| GFAP | ε2 | 0.115 [-0.120, 0.346] | .258 | | -0.097 [-0.214, 0.016] | | | | .116 |
|  | ε4 | -0.053 [-0.264, 0.158] | .349 | | 0.145 [0.074, 0.214] | | | | .005** |
|  |  | **European** | |  | | |  | |  |
|  |  | ***APOE* × Ancestry Interaction** | | | | ***APOE* Main Effect** | | |  |
| ATN(I) biomarker | ***APOE* allele** | **Estimate [95% CI]** | | ***P* value** | | **Estimate [95% CI]** | | | ***P* value** |
| Aꞵ42/40 | ε2 | 0.024 [-0.267, 0.319] | | .447 | | -0.005 [-0.198, 0.190] | | | .487 |
|  | ε4 | -0.112 [-0.293, 0.059] | | .195 | | -0.172 [-0.284, -0.063] | | | .018* |
| pTau-181 | ε2 | 0.155 [-0.119, 0.424] | | .181 | | -0.082 [-0.268, 0.102] | | | .260 |
|  | ε4 | 0.169 [-0.052, 0.395] | | .089 | | 0.155 [0.028, 0.268] | | | .032* |
| NfL | ε2 | -0.204 [-0.447, 0.051] | | .109 | | 0.057 [-0.140, 0.255] | | | .299 |
|  | ε4 | 0.126 [-0.041, 0.294] | | .153 | | -0.061 [-0.153, 0.037] | | | .214 |
| GFAP | ε2 | -0.064 [-0.297, 0.165] | | .341 | | -0.014 [-0.181, 0.153] | | | .458 |
|  | ε4 | 0.079 [-0.074, 0.231] | | .259 | | 0.092 [-0.009, 0.198] | | | .120 |
|  |  | **Amerindian** | |  | | |  | |  |
|  |  | ***APOE* × Ancestry Interaction** | | | | ***APOE* Main Effect** | | |  |
| ATN(I) biomarker | ***APOE* allele** | **Estimate [95% CI]** | | ***P* value** | | **Estimate [95% CI]** | | | ***P* value** |
| Aꞵ42/40 | ε2 | -0.405 [-1.034, 0.224] | | .110 | | 0.039 [-0.086, 0.172] | | | .300 |
|  | ε4 | 0.053 [-0.138, 0.241] | | .358 | | -0.250 [-0.336, -0.155] | | | <.001*** |
| pTau-181 | ε2 | 0.121 [-0.381, 0.621] | | .341 | | -0.005 [-0.133, 0.123] | | | .482 |
|  | ε4 | -0.107 [-0.339, 0.129] | | .232 | | 0.254 [0.141, 0.381] | | | <.001*** |
| NfL | ε2 | 0.345 [-0.120, 0.792] | | .129 | | -0.080 [-0.179, 0.023] | | | .120 |
|  | ε4 | -0.077 [-0.268, 0.114] | | .292 | | 0.028 [-0.061, 0.117] | | | .307 |
| GFAP | ε2 | -0.198 [-0.708, 0.329] | | .259 | | -0.033 [-0.125, 0.059] | | | .320 |
|  | ε4 | -0.064 [-0.264, 0.133] | | .315 | | 0.153 [0.059, 0.247] | | | .005** |

NOTE: Models were adjusted for age, sex, study center, and global genetic ancestry proportions (African and Amerindian, with European as the reference). Models are based on the additive inheritance mode with *APOE* ε3 used as the reference allele. Effect sizes were estimated using the complex survey design method, standardized to the survey-weighted SD of each biomarker in the overall cohort, and reported with 95% CIs. *P* values were estimated based on permutation testing with 10,000 permutations. Asterisks indicate statistical significance: ****P* < .001, ***P* < .01, **P* < .05.

**Supplementary figure 6. Interaction of local genetic ancestry counts and *APOE* ε2 allele on ATN(I) biomarkers.**


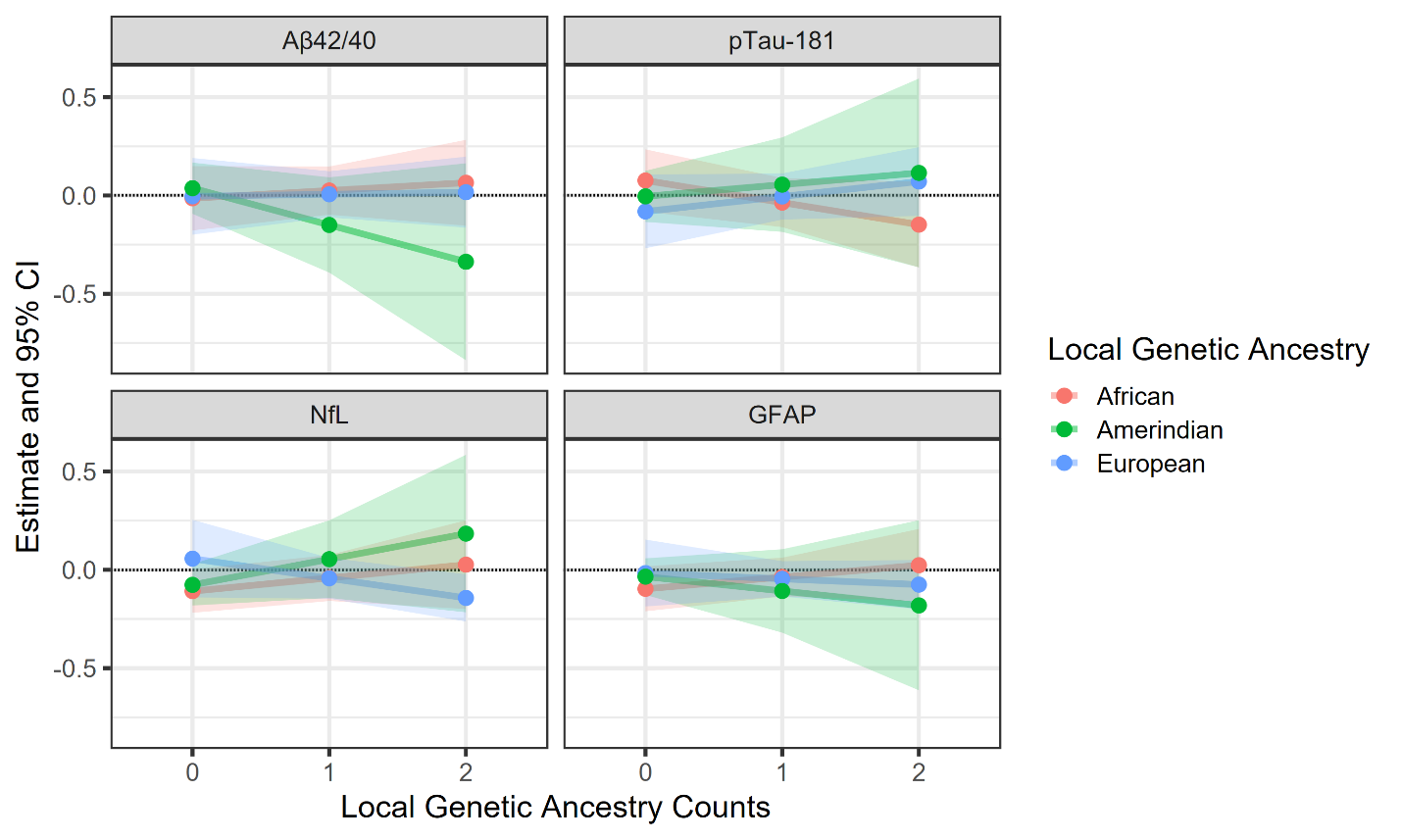


NOTE: Associations were tested at ancestry counts of 0, 1, and 2, with effect estimates represented by points and shaded areas indicating 95% confidence intervals. Estimates were derived from linear combinations of regression coefficients, adjusting for age, sex, study center, and global genetic ancestry proportions (African and Amerindian, with European as reference), and were standardized to the survey-weighted SD of each biomarker in the overall cohort.

**Supplementary table S11. Stratified interaction of local ancestry proportion and *APOE* alleles on ATN(I) biomarkers.**

|  |  | Aβ42/40 |  | |  | |  |
| --- | --- | --- | --- | --- | --- | --- | --- |
|  |  | ***APOE* ε2** |  | ***APOE* ε4** | | |  |
| **Ancestry** | **Count** | **Estimate [95% CI]** | ***P* value** | **Estimate [95% CI]** | | | ***P* value** |
| **African** | 0 | -0.014 [-0.178, 0.149] | .862 | -0.258 [-0.345, -0.171] | | | <.001*** |
|  | 1 | 0.025 [-0.097, 0.147] | .680 | -0.201 [-0.296, -0.106] | | | <.001*** |
|  | 2 | 0.065 [-0.153, 0.283] | .553 | -0.143 [-0.325, 0.038] | | | .114 |
| **Amerindian** | 0 | 0.037 [-0.093, 0.167] | .568 | -0.248 [-0.339, -0.158] | | | <.001*** |
|  | 1 | -0.150 [-0.392, 0.092] | .215 | -0.218 [-0.302, -0.135] | | | <.001*** |
|  | 2 | -0.337 [-0.838, 0.164] | .178 | -0.189 [-0.347, -0.031] | | | .016* |
| **European** | 0 | -0.004 [-0.199, 0.191] | .966 | -0.175 [-0.288, -0.063] | | | .002** |
|  | 1 | 0.006 [-0.111, 0.123] | .915 | -0.231 [-0.303, -0.159] | | | <.001*** |
|  | 2 | 0.017 [-0.164, 0.197] | .854 | -0.287 [-0.403, -0.170] | | | <.001*** |
|  |  | **pTau-181** |  | |  |  | |
|  |  | ***APOE* ε2** |  | ***APOE* ε4** | |  | |
| **Ancestry** | **Count** | **Estimate [95% CI]** | ***P* value** | **Estimate [95% CI]** | | ***P* value** | |
| **African** | 0 | 0.076 [-0.082, 0.234] | .335 | 0.263 [0.153, 0.373] | | <.001*** | |
|  | 1 | -0.036 [-0.161, 0.089] | .564 | 0.203 [0.096, 0.311] | | <.001*** | |
|  | 2 | -0.148 [-0.366, 0.069] | .173 | 0.145 [-0.062, 0.352] | | .160 | |
| **Amerindian** | 0 | -0.004 [-0.134, 0.125] | .946 | 0.261 [0.143, 0.379] | | <.001*** | |
|  | 1 | 0.055 [-0.185, 0.295] | .646 | 0.206 [0.114, 0.298] | | <.001*** | |
|  | 2 | 0.115 [-0.366, 0.595] | .632 | 0.151 [-0.027, 0.329] | | .089 | |
| **European** | 0 | -0.081 [-0.268, 0.106] | .386 | 0.150 [0.026, 0.274] | | .015* | |
|  | 1 | -0.005 [-0.122, 0.112] | .932 | 0.234 [0.147, 0.321] | | <.001*** | |
|  | 2 | 0.071 [-0.104, 0.246] | .416 | 0.318 [0.161, 0.475] | | <.001*** | |
|  |  | **NfL** |  | |  |  | |
|  |  | ***APOE* ε2** |  | ***APOE* ε4** | |  | |
| **Ancestry** | **Count** | **Estimate [95% CI]** | ***P* value** | **Estimate [95% CI]** | | ***P* value** | |
| **African** | 0 | -0.109 [-0.218, 0.002] | .049* | 0.028 [-0.062, 0.119] | | .530 | |
|  | 1 | -0.041 [-0.158, 0.076] | .481 | -0.016 [-0.102, 0.070] | | .709 | |
|  | 2 | 0.026 [-0.199, 0.252] | .816 | -0.061 [-0.226, 0.104] | | .463 | |
| **Amerindian** | 0 | -0.077 [-0.181, 0.029] | .147 | 0.028 [-0.063, 0.119] | | .541 | |
|  | 1 | 0.054 [-0.143, 0.252] | .584 | -0.009 [-0.090, 0.072] | | .824 | |
|  | 2 | 0.184 [-0.216, 0.585] | .357 | -0.046 [-0.199, 0.108] | | .550 | |
| **European** | 0 | 0.057 [-0.140, 0.254] | .567 | -0.061 [-0.161, 0.039] | | .222 | |
|  | 1 | -0.043 [-0.147, 0.061] | .405 | 0.002 [-0.068, 0.073] | | .945 | |
|  | 2 | -0.143 [-0.263, -0.022] | .018* | 0.066 [-0.055, 0.186] | | .276 | |

|  |  | GFAP |  | |  |  |
| --- | --- | --- | --- | --- | --- | --- |
|  |  | ***APOE* ε2** |  | ***APOE* ε4** | |  |
| **Ancestry** | **Count** | **Estimate [95% CI]** | ***P* value** | **Estimate [95% CI]** | | ***P* value** |
| **African** | 0 | -0.097 [-0.211, 0.018] | .091 | 0.144 [0.072, 0.216] | | <.001*** |
|  | 1 | -0.037 [-0.135, 0.062] | .455 | 0.120 [0.021, 0.219] | | .015* |
|  | 2 | 0.023 [-0.160, 0.208] | .799 | 0.097 [-0.097, 0.290] | | .319 |
| **Amerindian** | 0 | -0.035 [-0.128, 0.058] | .454 | 0.153 [0.058, 0.247] | | .001** |
|  | 1 | -0.108 [-0.320, 0.105] | .310 | 0.121 [0.051, 0.191] | | <.001*** |
|  | 2 | -0.181 [-0.613, 0.252] | .404 | 0.090 [-0.055, 0.234] | | .216 |
| **European** | 0 | -0.017 [-0.186, 0.153] | .843 | 0.092 [-0.011, 0.194] | | .073 |
|  | 1 | -0.046 [-0.136, 0.044] | .306 | 0.132 [0.065, 0.199] | | <.001*** |
|  | 2 | -0.075 [-0.201, 0.050] | .229 | 0.171 [0.069, 0.275] | | <.001*** |

NOTE: Estimates (in SD units) are derived from linear combinations of regression coefficients, adjusting for age, sex, study center, and global genetic ancestry proportions (African and Amerindian, with European as reference). Asterisks indicate statistical significance: ****P* < .001, ***P* < .01, **P* < .05.

**Supplementary figure 7. Associations between ATN(I) biomarkers and APOE allele by global genetic ancestry, stratified by age.**


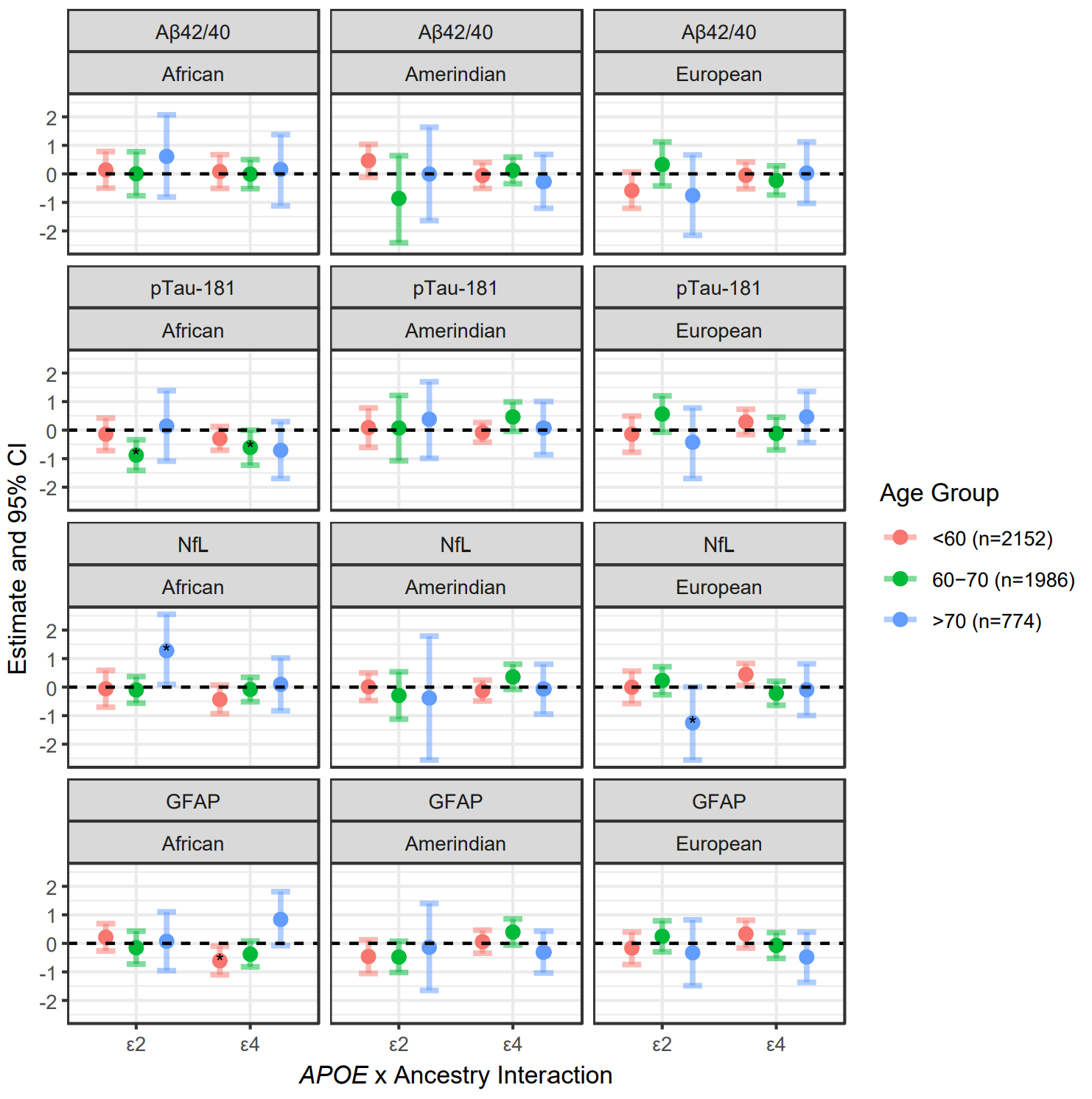


NOTE: Models were adjusted for sex and study center. Models are based on the additive inheritance mode with *APOE* ε3 used as the reference allele. Effect sizes were estimated using the complex survey design method, standardized to the survey-weighted SD of each biomarker in the overall cohort, and reported with 95% CIs. *P* values were estimated based on permutation testing with 10,000 permutations. Asterisks indicate statistical significance: ****P* < .001, ***P* < .01, **P* < .05.

| < 60 (n = 2152) | | | | | | |
| --- | --- | --- | --- | --- | --- | --- |
|  | **African** |  | **European** |  | **Amerindian** |  |
| ***APOE* ε2 × Ancestry Interaction** | | | | | | |
| **Biomarker** | **Estimate [95% CI]** | ***P* value** | **Estimate [95% CI]** | ***P* value** | **Estimate [95% CI]** | ***P* value** |
| Aꞵ42/40 | 0.138 [-0.500, 0.784] | .379 | -0.586 [-1.207, 0.066] | .082 | 0.466 [-0.121, 1.034] | .158 |
| pTau-181 | -0.141 [-0.720, 0.424] | .368 | -0.140 [-0.777, 0.494] | .357 | 0.089 [-0.607, 0.777] | .419 |
| NfL | -0.060 [-0.702, 0.587] | .443 | -0.004 [-0.575, 0.562] | .497 | 0.009 [-0.473, 0.498] | .482 |
| GFAP | 0.214 [-0.264, 0.692] | .274 | -0.165 [-0.741, 0.395] | .331 | -0.461 [-1.054, 0.125] | .134 |
| ***APOE* ε4 × Ancestry Interaction** | | | | | | |
| **Biomarker** | **Estimate [95% CI]** | ***P* value** | **Estimate [95% CI]** | ***P* value** | **Estimate [95% CI]** | ***P* value** |
| Aꞵ42/40 | 0.082 [-0.509, 0.672] | .412 | -0.053 [-0.526, 0.414] | .451 | -0.056 [-0.509, 0.397] | .435 |
| pTau-181 | -0.297 [-0.706, 0.129] | .206 | 0.282 [-0.155, 0.734] | .214 | -0.079 [-0.424, 0.268] | .422 |
| NfL | -0.434 [-0.932, 0.073] | .111 | 0.447 [0.063, 0.830] | .097 | -0.126 [-0.485, 0.243] | .367 |
| GFAP | -0.610 [-1.104, -0.102] | .032* | 0.329 [-0.165, 0.807] | .159 | 0.058 [-0.346, 0.461] | .422 |
| **60 – 70 (n = 1986)** | | | | | | |
|  | **African** |  | **European** |  | **Amerindian** |  |
| ***APOE* ε2 × Ancestry Interaction** | | | | | | |
| **Biomarker** | **Estimate [95% CI]** | ***P* value** | **Estimate [95% CI]** | ***P* value** | **Estimate [95% CI]** | ***P* value** |
| Aꞵ42/40 | 0.003 [-0.767, 0.776] | .514 | 0.328 [-0.422, 1.121] | .245 | -0.862 [-2.414, 0.638] | .090 |
| pTau-181 | -0.876 [-1.412, -0.339] | .030* | 0.565 [-0.072, 1.201] | .112 | 0.075 [-1.073, 1.215] | .425 |
| NfL | -0.100 [-0.562, 0.370] | .425 | 0.230 [-0.268, 0.715] | .304 | -0.294 [-1.124, 0.536] | .326 |
| GFAP | -0.152 [-0.725, 0.428] | .376 | 0.247 [-0.297, 0.791] | .276 | -0.478 [-1.021, 0.072] | .206 |
| ***APOE* ε4 × Ancestry Interaction** | | | | | | |
| **Biomarker** | **Estimate [95% CI]** | ***P* value** | **Estimate [95% CI]** | ***P* value** | **Estimate [95% CI]** | ***P* value** |
| Aꞵ42/40 | -0.009 [-0.517, 0.500] | .486 | -0.233 [-0.741, 0.284] | .255 | 0.121 [-0.345, 0.586] | .374 |
| pTau-181 | -0.607 [-1.229, 0.000] | .050* | -0.117 [-0.692, 0.452] | .367 | 0.466 [-0.042, 0.989] | .101 |
| NfL | -0.080 [-0.511, 0.345] | .408 | -0.217 [-0.639, 0.204] | .254 | 0.358 [-0.080, 0.805] | .151 |
| GFAP | -0.379 [-0.824, 0.082] | .141 | -0.072 [-0.527, 0.379] | .410 | 0.395 [-0.061, 0.857] | .133 |
| **> 70 (n = 774)** | | | | | | |
|  | **African** |  | **European** |  | **Amerindian** |  |
| ***APOE* ε2 × Ancestry Interaction** | | | | | | |
| **Biomarker** | **Estimate [95% CI]** | ***P* value** | **Estimate [95% CI]** | ***P* value** | **Estimate [95% CI]** | ***P* value** |
| Aꞵ42/40 | 0.612 [-0.810, 2.069] | .214 | -0.759 [-2.155, 0.664] | .162 | -0.006 [-1.638, 1.638] | .480 |
| pTau-181 | 0.141 [-1.088, 1.384] | .407 | -0.424 [-1.695, 0.777] | .290 | 0.381 [-0.989, 1.695] | .328 |
| NfL | 1.277 [0.096, 2.554] | .048* | -1.252 [-2.554, 0.008] | .041* | -0.383 [-2.554, 1.788] | .324 |
| GFAP | 0.074 [-0.956, 1.104] | .455 | -0.329 [-1.483, 0.824] | .316 | -0.137 [-1.647, 1.400] | .426 |
| ***APOE* ε4 × Ancestry Interaction** | | | | | | |
| **Biomarker** | **Estimate [95% CI]** | ***P* value** | **Estimate [95% CI]** | ***P* value** | **Estimate [95% CI]** | ***P* value** |
| Aꞵ42/40 | 0.155 [-1.121, 1.379] | .409 | 0.028 [-1.034, 1.121] | .489 | -0.276 [-1.207, 0.681] | .325 |
| pTau-181 | -0.706 [-1.695, 0.297] | .123 | 0.466 [-0.438, 1.356] | .214 | 0.075 [-0.862, 1.003] | .441 |
| NfL | 0.097 [-0.830, 1.022] | .425 | -0.093 [-0.996, 0.817] | .439 | -0.066 [-0.945, 0.805] | .461 |
| GFAP | 0.840 [-0.079, 1.812] | .081 | -0.478 [-1.367, 0.395] | .202 | -0.313 [-1.038, 0.428] | .296 |

**Supplementary table S12. Associations between ATN(I) biomarkers and *APOE* allele by global genetic ancestry, stratified by age.**

NOTE: Models were adjusted for sex and study center. Models are based on the additive inheritance mode with *APOE* ε3 used as the reference allele. Effect sizes were estimated using the complex survey design method, standardized to the survey-weighted SD of each biomarker in the overall cohort, and reported with 95% CIs. *P* values were estimated based on permutation testing with 10,000 permutations. Asterisks indicate statistical significance: ****P* < .001, ***P* < .01, **P* < .05.

**Supplementary figure 8. Associations between ATN(I) biomarkers and *APOE* allele by global genetic ancestry, stratified by genetic analysis group.**


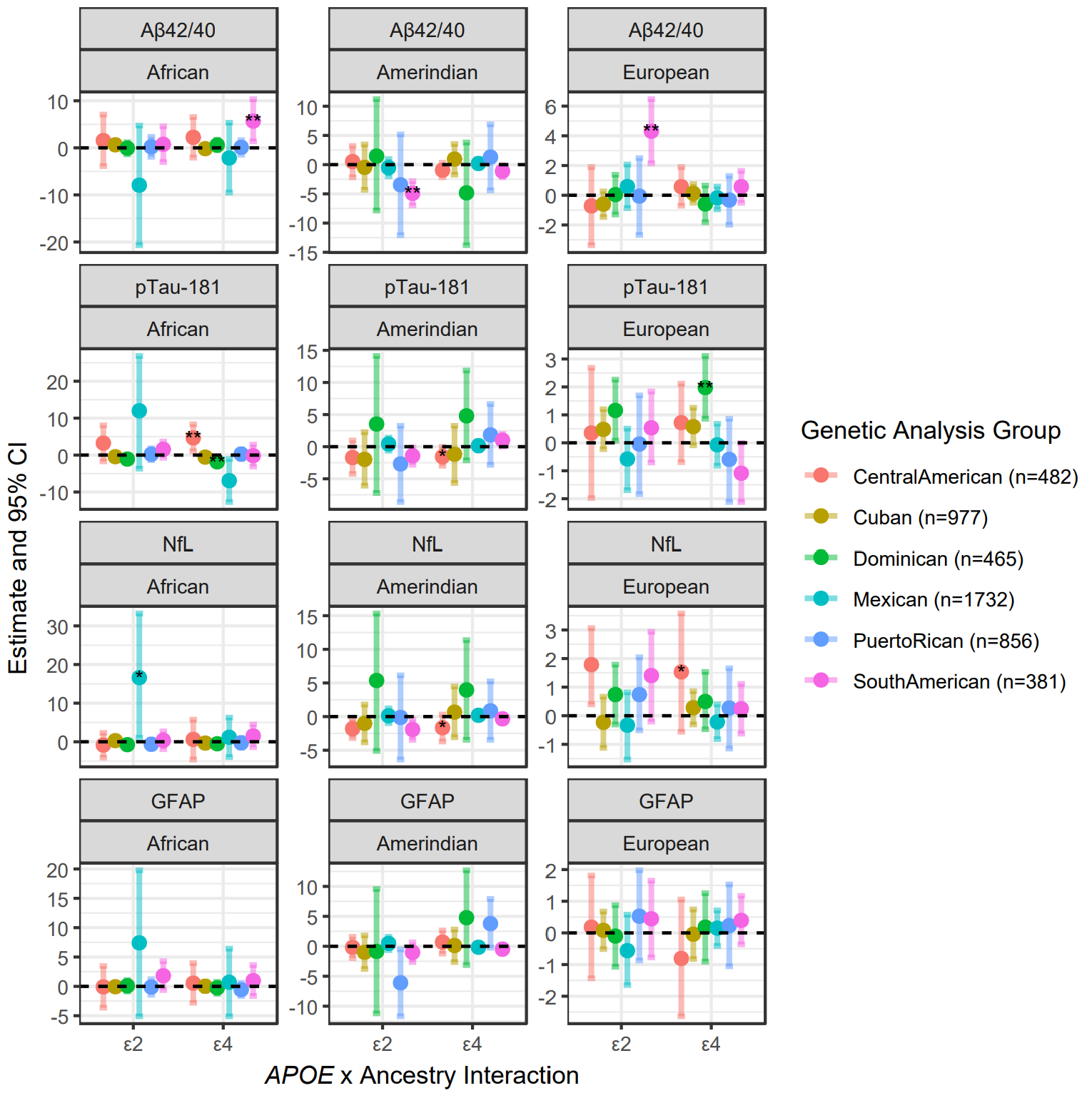


NOTE: Models were adjusted for age, sex, and study center. Models are based on the additive inheritance mode with *APOE* ε3 used as the reference allele. Effect sizes were estimated using the complex survey design method, standardized to the survey-weighted SD of each biomarker in the overall cohort, and reported with 95% CIs. *P* values were estimated based on permutation testing with 10,000 permutations. Asterisks indicate statistical significance: ****P* < .001, ***P* < .01, **P* < .05.

**Supplementary table S13. Associations between ATN(I) biomarkers and *APOE* allele by global genetic ancestry, stratified by genetic analysis group.**

| Central American (n = 482) | | | | | | | | | | | | |  |  |
| --- | --- | --- | --- | --- | --- | --- | --- | --- | --- | --- | --- | --- | --- | --- |
|  | **African** | |  | | **European** | |  | | **Amerindian** | |  | |  |  |
| ***APOE* ε2 × Ancestry Interaction** | | | | | | | | | | | | |  |  |
| **Biomarker** | **Estimate [95% CI]** | | ***P* value** | | **Estimate [95% CI]** | | ***P* value** | | **Estimate [95% CI]** | | ***P* value** | |  |  |
| Aꞵ42/40 | 1.552 [-3.879, 7.069] | | .322 | | -0.716 [-3.362, 1.897] | | .334 | | 0.526 [-2.155, 3.190] | | .378 | |  |  |
| pTau-181 | 3.249 [-1.695, 8.051] | | .170 | | 0.353 [-1.977, 2.684] | | .381 | | -1.695 [-4.237, 0.975] | | .151 | |  |  |
| NfL | -0.843 [-4.087, 2.299] | | .383 | | 1.788 [0.396, 3.065] | | .129 | | -1.788 [-3.193, -0.383] | | .126 | |  |  |
| GFAP | -0.084 [-3.624, 3.460] | | .482 | | 0.181 [-1.433, 1.812] | | .432 | | -0.181 [-1.977, 1.582] | | .428 | |  |  |
| ***APOE* ε4 × Ancestry Interaction** | | | | | | | | | | | | |  |  |
| **Biomarker** | **Estimate [95% CI]** | | ***P* value** | | **Estimate [95% CI]** | | ***P* value** | | **Estimate [95% CI]** | | ***P* value** | |  |  |
| Aꞵ42/40 | 2.241 [-2.069, 6.552] | | .129 | | 0.578 [-0.707, 1.897] | | .241 | | -0.948 [-2.155, 0.336] | | .151 | |  |  |
| pTau-181 | 4.661 [1.031, 8.333] | | .009** | | 0.720 [-0.692, 2.119] | | .182 | | -1.554 [-2.966, -0.037] | | .038* | |  |  |
| NfL | 0.613 [-4.598, 5.747] | | .366 | | 1.533 [-0.562, 3.576] | | .033* | | -1.660 [-3.704, 0.307] | | .026* | |  |  |
| GFAP | 0.544 [-2.801, 3.954] | | .383 | | -0.807 [-2.636, 1.054] | | .148 | | 0.708 [-1.219, 2.636] | | .199 | |  |  |
| **Cuban (n = 977)** | | | | | | | | | | | | |  |  |
|  | **African** | |  | | **European** | |  | | **Amerindian** | |  | |  |  |
| ***APOE* ε2 × Ancestry Interaction** | | | | | | | | | | | | |  |  |
| **Biomarker** | **Estimate [95% CI]** | | ***P* value** | | **Estimate [95% CI]** | | ***P* value** | | **Estimate [95% CI]** | | ***P* value** | |  |  |
| Aꞵ42/40 | 0.647 [-0.224, 1.552] | | .123 | | -0.595 [-1.466, 0.259] | | .135 | | -0.466 [-4.310, 3.448] | | .422 | |  |  |
| pTau-181 | -0.438 [-1.172, 0.297] | | .211 | | 0.480 [-0.226, 1.201] | | .187 | | -1.977 [-6.073, 2.260] | | .211 | |  |  |
| NfL | 0.294 [-0.702, 1.277] | | .282 | | -0.230 [-1.124, 0.664] | | .317 | | -1.009 [-3.831, 1.788] | | .342 | |  |  |
| GFAP | -0.063 [-0.659, 0.527] | | .465 | | 0.081 [-0.511, 0.675] | | .441 | | -1.005 [-3.789, 1.812] | | .338 | |  |  |
| ***APOE* ε4 × Ancestry Interaction** | | | | | | | | | | | | |  |  |
| **Biomarker** | **Estimate [95% CI]** | | ***P* value** | | **Estimate [95% CI]** | | ***P* value** | | **Estimate [95% CI]** | | ***P* value** | |  |  |
| Aꞵ42/40 | -0.172 [-0.810, 0.457] | | .354 | | 0.129 [-0.500, 0.750] | | .373 | | 0.948 [-1.724, 3.534] | | .322 | |  |  |
| pTau-181 | -0.551 [-1.257, 0.141] | | .101 | | 0.579 [-0.090, 1.257] | | .091 | | -1.172 [-5.650, 3.249] | | .283 | |  |  |
| NfL | -0.332 [-0.945, 0.281] | | .219 | | 0.281 [-0.307, 0.856] | | .253 | | 0.664 [-3.065, 4.470] | | .358 | |  |  |
| GFAP | 0.018 [-0.791, 0.824] | | .478 | | -0.041 [-0.824, 0.741] | | .460 | | 0.129 [-2.636, 2.801] | | .472 | |  |  |
| **Dominican (n = 465)** | | | | | | | | | | | | |  |  |
|  | **African** | |  | | **European** | |  | | **Amerindian** | |  | |  |  |
| ***APOE* ε2 × Ancestry Interaction** | | | | | | | | | | | | |  |  |
| **Biomarker** | **Estimate [95% CI]** | | ***P* value** | | **Estimate [95% CI]** | | ***P* value** | | **Estimate [95% CI]** | | ***P* value** | |  |  |
| Aꞵ42/40 | -0.062 [-1.379, 1.207] | | .465 | | 0.044 [-1.293, 1.379] | | .472 | | 1.466 [-7.845, 11.207] | | .414 | |  |  |
| pTau-181 | -1.073 [-2.119, -0.025] | | .089 | | 1.158 [0.079, 2.260] | | .096 | | 3.531 [-7.203, 14.124] | | .282 | |  |  |
| NfL | -0.741 [-1.788, 0.281] | | .169 | | 0.741 [-0.307, 1.788] | | .188 | | 5.364 [-5.109, 15.326] | | .178 | |  |  |
| GFAP | 0.097 [-0.873, 1.071] | | .440 | | -0.100 [-1.071, 0.873] | | .442 | | -0.857 [-11.203, 9.555] | | .450 | |  |  |
| ***APOE* ε4 × Ancestry Interaction** | | | | | | | | | | | | |  |  |
| **Biomarker** | **Estimate [95% CI]** | | ***P* value** | | **Estimate [95% CI]** | | ***P* value** | | **Estimate [95% CI]** | | ***P* value** | |  |  |
| Aꞵ42/40 | 0.595 [-0.491, 1.724] | | .210 | | -0.586 [-1.810, 0.655] | | .240 | | -4.828 [-13.793, 3.793] | | .167 | |  |  |
| pTau-181 | -1.836 [-2.825, -0.763] | | .004** | | 1.977 [0.862, 3.107] | | .005** | | 4.802 [-2.119, 11.864] | | .166 | |  |  |
| NfL | -0.511 [-1.405, 0.370] | | .230 | | 0.498 [-0.473, 1.533] | | .259 | | 3.959 [-3.448, 11.367] | | .201 | |  |  |
| GFAP | -0.247 [-1.269, 0.774] | | .358 | | 0.181 [-0.906, 1.252] | | .408 | | 4.778 [-3.130, 12.685] | | .146 | |  |  |
|  |  | |  | |  | |  | |  | |  | |  |  |
| **Mexican (n = 1732)** | | | | | | | | | | | | |  |  |
|  | | | **African** | |  | | **European** | |  | | **Amerindian** |  |  |  |
| ***APOE* ε2 × Ancestry Interaction** | | | | | | | | | | | | |  |  |
| **Biomarker** | | | **Estimate [95% CI]** | | ***P* value** | | **Estimate [95% CI]** | | ***P* value** | | **Estimate [95% CI]** | ***P* value** |  |  |
| Aꞵ42/40 | | | -7.931 [-20.690, 4.741] | | .156 | | 0.586 [-0.862, 2.069] | | .259 | | -0.509 [-1.983, 0.948] | .287 |  |  |
| pTau-181 | | | 12.006 [-3.672, 26.836] | | .061 | | -0.579 [-1.695, 0.508] | | .261 | | 0.424 [-0.621, 1.412] | .324 |  |  |
| NfL | | | 16.603 [1.047, 33.206] | | .012* | | -0.332 [-1.533, 0.817] | | .350 | | 0.123 [-0.971, 1.226] | .451 |  |  |
| GFAP | | | 7.414 [-5.107, 19.769] | | .151 | | -0.560 [-1.647, 0.577] | | .251 | | 0.478 [-0.643, 1.582] | .291 |  |  |
| ***APOE* ε4 × Ancestry Interaction** | | | | | | | | | | | | | | |
| **Biomarker** | | | **Estimate [95% CI]** | | ***P* value** | | **Estimate [95% CI]** | | ***P* value** | | **Estimate [95% CI]** | | ***P* value** | |
| Aꞵ42/40 | | | -2.155 [-9.483, 5.345] | | .329 | | -0.172 [-0.948, 0.586] | | .398 | | 0.198 [-0.543, 0.948] | | .388 | |
| pTau-181 | | | -6.921 [-12.712, -1.073] | | .078 | | -0.072 [-0.819, 0.678] | | .437 | | 0.155 [-0.565, 0.876] | | .376 | |
| NfL | | | 1.162 [-3.959, 6.258] | | .420 | | -0.217 [-0.817, 0.396] | | .342 | | 0.192 [-0.409, 0.805] | | .349 | |
| GFAP | | | 0.692 [-5.107, 6.425] | | .452 | | 0.150 [-0.412, 0.708] | | .408 | | -0.160 [-0.708, 0.395] | | .402 | |
| **Puerto Rican (n = 856)** | | | | | | | | | | | | | | |
|  | | | **African** | |  | | **European** | |  | | **Amerindian** | |  | |
| ***APOE* ε2 × Ancestry Interaction** | | | | | | | | | | | | | | |
| **Biomarker** | | | **Estimate [95% CI]** | | ***P* value** | | **Estimate [95% CI]** | | ***P* value** | | **Estimate [95% CI]** | | ***P* value** | |
| Aꞵ42/40 | | | 0.216 [-1.897, 2.328] | | .430 | | -0.058 [-2.672, 2.500] | | .481 | | -3.448 [-12.069, 5.172] | | .241 | |
| pTau-181 | | | 0.226 [-1.356, 1.836] | | .421 | | -0.044 [-1.836, 1.695] | | .500 | | -2.684 [-8.616, 3.249] | | .282 | |
| NfL | | | -0.639 [-1.788, 0.549] | | .250 | | 0.741 [-0.511, 2.043] | | .253 | | -0.128 [-6.386, 6.130] | | .490 | |
| GFAP | | | -0.109 [-1.433, 1.219] | | .453 | | 0.527 [-0.873, 1.977] | | .309 | | -6.096 [-11.697, -0.527] | | .097 | |
| ***APOE* ε4 × Ancestry Interaction** | | | | | | | | | | | | | | |
| **Biomarker** | | | **Estimate [95% CI]** | | ***P* value** | | **Estimate [95% CI]** | | ***P* value** | | **Estimate [95% CI]** | | ***P* value** | |
| Aꞵ42/40 | | | 0.112 [-1.466, 1.638] | | .441 | | -0.319 [-1.983, 1.293] | | .362 | | 1.293 [-4.397, 6.897] | | .334 | |
| pTau-181 | | | 0.268 [-1.073, 1.554] | | .359 | | -0.593 [-2.119, 0.862] | | .251 | | 1.836 [-2.825, 6.638] | | .251 | |
| NfL | | | -0.281 [-1.533, 0.983] | | .360 | | 0.268 [-1.149, 1.660] | | .380 | | 0.856 [-3.448, 5.236] | | .367 | |
| GFAP | | | -0.560 [-1.647, 0.593] | | .228 | | 0.231 [-1.054, 1.532] | | .386 | | 3.789 [-0.247, 7.908] | | .090 | |
| **South American (n = 381)** | | | | | | | | | | | | | | |
|  | | | **African** | |  | | **European** | |  | | **Amerindian** | |  | |
| ***APOE* ε2 × Ancestry Interaction** | | | | | | | | | | | | | | |
| **Biomarker** | | | **Estimate [95% CI]** | | ***P* value** | | **Estimate [95% CI]** | | ***P* value** | | **Estimate [95% CI]** | | ***P* value** | |
| Aꞵ42/40 | | | 0.759 [-3.017, 4.569] | | .376 | | 4.310 [2.155, 6.466] | | .005** | | -4.914 [-6.983, -2.845] | | .002** | |
| pTau-181 | | | 1.554 [-0.734, 3.672] | | .230 | | 0.537 [-0.706, 1.836] | | .369 | | -1.412 [-2.825, -0.127] | | .165 | |
| NfL | | | 0.370 [-1.916, 2.682] | | .418 | | 1.405 [-0.204, 2.937] | | .157 | | -1.916 [-3.448, -0.434] | | .079 | |
| GFAP | | | 1.812 [-0.626, 4.283] | | .189 | | 0.445 [-0.774, 1.647] | | .395 | | -1.021 [-2.636, 0.643] | | .230 | |
| ***APOE* ε4 × Ancestry Interaction** | | | | | | | | | | | | | | |
| **Biomarker** | | | **Estimate [95% CI]** | | ***P* value** | | **Estimate [95% CI]** | | ***P* value** | | **Estimate [95% CI]** | | ***P* value** | |
| Aꞵ42/40 | | | 5.690 [1.379, 10.345] | | .009** | | 0.578 [-0.509, 1.638] | | .234 | | -1.121 [-2.155, -0.095] | | .065 | |
| pTau-181 | | | -0.155 [-3.107, 2.825] | | .499 | | -1.088 [-2.119, -0.009] | | .078 | | 1.017 [0.032, 1.977] | | .081 | |
| NfL | | | 1.533 [-1.405, 4.470] | | .231 | | 0.243 [-0.626, 1.111] | | .355 | | -0.332 [-0.971, 0.307] | | .312 | |
| GFAP | | | 0.972 [-1.631, 3.624] | | .315 | | 0.395 [-0.362, 1.170] | | .292 | | -0.478 [-1.252, 0.297] | | .234 | |

NOTE: Models were adjusted for age, sex, and study center. Models are based on the additive inheritance mode with *APOE* ε3 used as the reference allele. Effect sizes were estimated using the complex survey design method, standardized to the survey-weighted SD of each biomarker in the overall cohort, and reported with 95% CIs. *P* values were estimated based on permutation testing with 10,000 permutations. Asterisks indicate statistical significance: ****P* < .001, ***P* < .01, **P* < .05.

**Supplementary table S14. Comparisons of design and results of prior *APOE* and AD biomarker studies.**

| Study | Population | Sample size | Predictor | Covariates | Outcome | Effect size | Significance |
| --- | --- | --- | --- | --- | --- | --- | --- |
| [4] | Community-dwelling older adults (59% Black, 41% White) | 1,038 | ε4 carrier × GFAP (log_10_) |  | Cognitive decline | β = -0.07 | *P* = .02 |
|  |  |  | ε4 carrier × NfL (tertial 1 vs 2) |  | Cognitive decline | β = -0.04 | *P* = .006 |
| [5] | AIBL: CU, MCI, AD combined | 225 | ε4 carrier status | unadjusted | Plasma Aβ42/40 (log_e_) |  | *P* = .061 |
|  |  |  |  |  | Plasma pTau-181 |  | *P* < .001 |
|  |  |  |  |  | Plasma GFAP |  | *P* = .040 |
|  |  |  |  |  | Plasma NfL |  | *P* = .758 |
|  |  |  |  | age, sex, diagnosis, PET Aβ+ status | Plasma Aβ42/40 (log_e_) |  | *P* = .215 |
|  |  |  |  |  | Plasma pTau-181 |  | *P* = .178 |
|  |  |  |  |  | Plasma GFAP |  | *P* = .178 |
|  |  |  |  |  | Plasma NfL |  | *P* = .521 |
| [23] | WHICAP (Caribbean Hispanics) | 366 | ε4 carrier status |  | Plasma pTau-181 | β = 0.31 SD | *P* = .014 |
|  | HABS-HD (Mexican American) | 534 | ε4 carrier status |  | Plasma pTau-181 | β = 0.32 SD | *P* = .003 |
| [49] | non‐demented participants from eight population‐based studies | 12,369 | *APOE* rs429358 (ε4 minor allele) | sex, age, PCs | Plasma Aβ42/40 | β = - 0.212 SD | *P* = 6.46e-20 |
| [50] | cognitively healthy super‐seniors | 370 | ε4 carrier status | unadjusted  (Mann–Whitney test) | Plasma Aβ42/40 |  | *P* = .0430 |
|  |  |  |  |  | Plasma pTau-181 |  | *P* = .0068 |
|  |  |  |  |  | Plasma GFAP |  | *P* = .0457 |
|  |  |  |  |  | Plasma NfL |  | *P* = .1147 |
|  |  |  |  | sex, age, BMI (MLR) | Plasma Aβ42/40 | β = -0.0194 | *P* = .127 |
|  |  |  |  |  | Plasma pTau-181 | β = 0.0649 | *P* = .0129 |
|  |  |  |  |  | Plasma GFAP | β = 0.0362 | *P* = .1213 |
|  |  |  |  |  | Plasma NfL | β = 0.0356 | *P* = .103 |
| [51] | Arizona *APOE* Cohort, cognitively unimpaired | 543 | ε4 allelic dose (heterozygote vs noncarrier) | age, sex, education | Plasma NfL |  | *P* = .02 |
|  |  |  | ε4 allelic dose (homozygote vs noncarrier) | age, sex, education | Plasma NfL |  | *P* = .03 |
|  |  |  | ε4 allelic dose (hetero- vs homozygote) | age, sex, education | Plasma NfL |  | *P* > .05 |
| [52] | ADNI, non-demented: CU and MCI | 570 | ε4 carrier status | age, gender, years of education | Plasma NfL | β = 0.152 | *P* = .02 |
|  |  |  |  |  |  |  |  |
| [61] | ADNI, ARIC, and FHS cohorts | 9,642 | ε4 carrier status | sex, age, age^2^ | Plasma Aβ40 | β = -1.287 | *P* = .322 |
|  |  |  | ε4 carrier status | sex, age, age^2^ | Plasma Aβ42 | β = -1.903 | *P* = 2.18e-12 |
|  |  |  | ε2 carrier status | sex, age, age^2^ | Plasma Aβ40 | β = -2.091 | *P* = .193 |
|  |  |  | ε2 carrier status | sex, age, age^2^ | Plasma Aβ42 | β = -0.105 | *P* = .761 |
|  |  |  |  |  |  |  |  |
| [62] | KARVIAH | ~100 | ε4 carrier status | unadjusted | Plasma GFAP |  | *P* = .743 |
|  |  |  |  |  | Plasma pTau-181 |  | *P* = .213 |
|  |  |  |  |  | Plasma NfL |  | *P* = .208 |
|  |  |  |  | age, sex | Plasma GFAP |  | *P* = .806 |
|  |  |  |  |  | Plasma pTau-181 |  | *P* = .059 |
|  |  |  |  |  | Plasma NfL |  | *P* = .431 |
| [63] | ADNI: CU, MCI, and AD | 714 | *APOE* rs769449 | age, gender, CDR-SB, top 3 PCs | Plasma pTau-181 | β = 3.361 | *P* = 6.26e-8 |
| [64] | HABS-HD (Mexican Americans and NHWs) | 1,577 | ε4 carrier status | Age, gender, education, race, MMSE, ε4*×*Race | Plasma Aβ42/40 | β = -0.0085 |  |
|  |  |  |  |  | Plasma pTau-181 | β = 1.07 |  |
|  |  |  |  |  | Plasma NfL | β = 6.361 |  |
|  |  |  | ε4*×*Race | Age, gender, education, race, MMSE, ε4 carrier status | Plasma Aβ42/40 | β = 0.0036 | *P* = .0215 |
|  |  |  |  |  | Plasma pTau-181 | β = -0.48 | *P* = .001 |
|  |  |  |  |  | Plasma NfL | β = -4.720 | *P* = .080 |
|  | HABS-HD (Mexican Americans only) | 792 | ε4 carrier status | Age, gender, education, MMSE | Plasma Aβ42/40 | β = -0.0014 | *P* = .2493 |
|  |  |  |  |  | Plasma pTau-181 | β = 0.1318 | *P* = .2076 |
|  |  |  |  |  | Plasma NfL | β = -2.9718 | *P* = .1506 |
| [65] | ADNI: normal controls (no MCI or AD) | 588 | ε4 carrier status | age, sex (Two-Way ANCOVA) | CSF Aβ42/40 |  | *P* < .001 |
|  |  |  |  |  | CSF pTau-181 |  | *P* < .001 |
|  |  |  |  |  | CSF GFAP |  | insignificant |
|  |  |  |  |  | CSF NfL |  | insignificant |
| [66] | ADNI: non-dementia (95.5% non-Hispanic) | 727 (plasma); 661 (CSF) | ε4 carrier status | unadjusted | CSF pTau-181 | t = 8.46 | *P* < .001 |
|  |  |  |  |  | Plasma pTau-181 | t = 5.81 | *P* < .001 |
|  |  |  |  | Age, cognitive classification | CSF pTau-181 | t = 8.51 | *P* < .001 |
|  |  |  |  |  | Plasma pTau-181 | t = 5.90 | *P* < .001 |
|  |  |  |  | Amyloid PET levels | CSF pTau-181 | t = 3.54 | *P* < .001 |
|  |  |  |  |  | Plasma pTau-181 | t = 2.36 | *P* = .02 |
